# Multiplexed Detection of Analytes on Single Test Strips with Antibody‐Gated Indicator‐Releasing Mesoporous Nanoparticles

**DOI:** 10.1002/anie.202009000

**Published:** 2020-10-01

**Authors:** Estela Climent, Mustafa Biyikal, Delia Gröninger, Michael G. Weller, Ramón Martínez‐Máñez, Knut Rurack

**Affiliations:** ^1^ Bundesanstalt für Materialforschung und -prüfung (BAM) Richard-Willstätter-Str. 11 12489 Berlin Germany; ^2^ Instituto Interuniversitario de Investigación de Reconocimiento Molecular y Desarrollo Tecnológico (IDM) Universitat Politècnica de València Universitat de València Camino de Vera, s/n 46022 Valencia Spain; ^3^ Unidad Mixta UPV-CIPF de Investigación en Mecanismos de Enfermedades y Nanomedicina Universitat Politècnica de València Centro de Investigación Príncipe Felipe C/ Eduardo Primo Yúfera 3 46012 Valencia Spain; ^4^ CIBER de Bioingeniería Biomateriales y Nanomedicina (CIBER-BBN) Spain

**Keywords:** explosives, hybrid materials, multiplexing, signal amplification, test-strip analysis

## Abstract

Rapid testing methods for the use directly at a point of need are expected to unfold their true potential especially when offering adequate capabilities for the simultaneous measurement of multiple analytes of interest. Considering the unique modularity, high sensitivity, and selectivity of antibody‐gated indicator delivery (gAID) systems, a multiplexed assay for three small‐molecule explosives (TATP, TNT, PETN) was thus developed, allowing to detect the analytes simultaneously with a single test strip at lower ppb concentrations in the liquid phase in <5 min using a fluorescence reader or a smartphone for readout. While the TNT and PETN systems were newly developed here, all the three systems also tolerated harsher matrices than buffered aqueous model solutions. Besides a single‐track strip, the outstanding modularity of the hybrid biosensor materials in combination with strip‐patterning technologies allowed us to obtain a multichannel strip in a straightforward manner, offering comparable analytical performance while allowing to be tailored even more to the user's need.

## Graphical abstract

The three small‐molecule explosives TATP, TNT and PETN can be detected simultaneously with a single test strip at lower ppb concentrations in the liquid phase in <5 min through the employment of tailored immunochemically gated hybrid materials using mobile devices for readout.

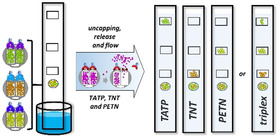

## Introduction

In areas such as health,^[1]^ food,^[2]^ security,^[3]^ or the environment,^[4]^ rapid tests for the use outside of a laboratory directly at a point of need are becoming increasingly important. Especially in view of the WHO's ASSURED principle,[^5^, ^6^] simple microfluidic chips or paper‐based devices possess an outstanding relevance in this regard.^[7]^ Besides robustness, user‐friendliness and analytical performance, their acceptance, popularity and breakthrough are determined by the capability to measure multiple analytes of interest simultaneously.^[8]^ Having to carry and use, for instance, three tests when needing to screen for three different analytes thwarts the basic idea of rapid and ASSURED testing. The implementation of multiplexing features while retaining simplicity, performance and portability is thus one of the prominent challenges in the field. Although multiplexed detection has been realized on paper, the majority of reports pertains to either proteinaceous biomarkers or metal ions.^[9]^ Examples for small organic molecules are scarce and have been realized in few‐lines pregnancy test‐type lateral flow assay formats, restricting modularity because only one type of optical reporter, gold nanoparticles, is used.^[10]^

**Scheme 1:**
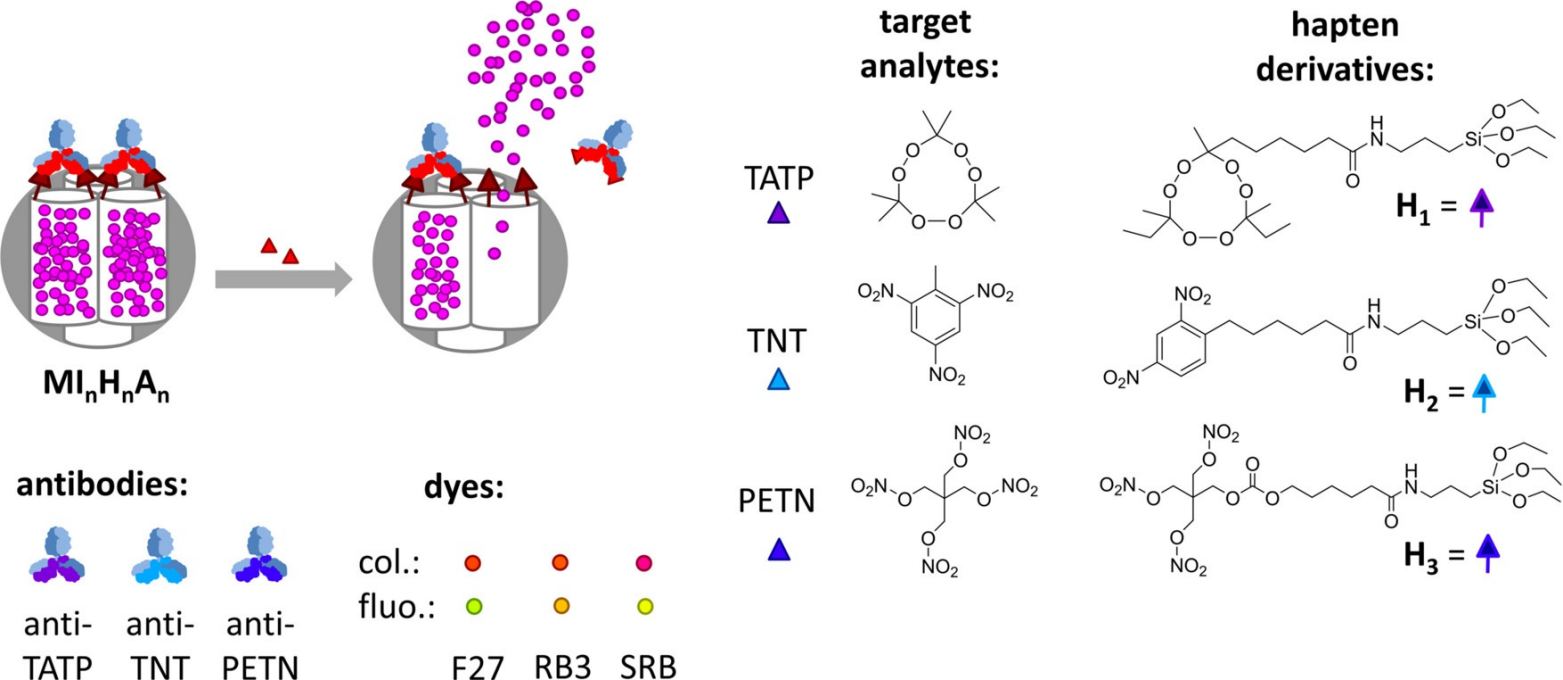
Architecture and principle of operation of the gAID systems **MI_n_H_n_A_n_** (upper left, arbitrary color code), chemical structures of corresponding target analyte/hapten pairs (right), polyclonal antibodies (lower far left: anti‐TATP=**A_1_**, anti‐TNT=**A_2_**, anti‐PETN=**A_3_**) and indicator dyes used (lower middle left: 2,7‐dichlorofluorescein, F27=**I_1_**; ruthenium tris(bipyridine), RB3=**I_2_**; sulforhodamine B, SRB=**I_3_**). Color codes of target analytes, hapten derivatives and antibodies used throughout the paper; for the dyes, the dots in the upper row (red, red, pink) represent the color of their solutions and the dots in the lower row (green, orange, yellow) represent their true emission colors.

Antibody‐gated indicator delivery (gAID) systems are an attractive alternative in terms of versatility and modularity.^[11]^ They constitute homogeneous immunoassays for which indication is decoupled from antigen‐antibody interaction, thus avoiding the use of reagent labelling. System architecture commonly comprises a mesoporous silica nanoparticle (MSN) scaffold that is loaded with indicator molecules and equipped with a dedicated gatekeeping/capping chemistry at the pore openings that ensures residence of the cargo in the pores in the absence of an analyte. In antibody‐gated systems, the gatekeeping/capping chemistry is commonly built up of a hapten derivative (the so‐called “gatekeeper”) grafted to the outer particle surface that binds to the respective antibody, acting as the “cap” (Scheme 1). Because a large number of indicator molecules can be released when a single analyte molecule binds to an antibody cap, thus inhibiting rebinding of the antibody to a grafted hapten derivative and allowing diffusion of the indicators out of the pores, such systems show intrinsic features of chemical signal amplification.^[12]^ When integrating these hybrid sensory materials on strips and employing them in lateral flow assay formats, simple yet powerful dipstick tests can result.^[13]^ The modularity of the design, as illustrated in Scheme 1, directly hints at the potential of the approach for multiplexed analysis. A first batch of MSNs can be loaded with a first type of indicator and closed with a first type of gatekeeping/capping immunochemistry, a second batch of MSNs can be loaded with a second type of indicator and closed with a second type of gatekeeping/capping immunochemistry, and so forth, potentially resulting in a library for which release of a specific type of indicator would directly transduce the presence of an analyte that has interacted selectively with the corresponding capping biomacromolecules.^[14]^ Here, we report on the first example of using this uniquely generic biomimetic strategy for the simultaneous detection of three small organic molecules on a single test strip with high sensitivity and selectivity. The three analytes chosen were the explosives triacetone triperoxide (TATP), trinitrotoluene (TNT) and nitropenta (PETN), the relevance of which is obvious, especially for security reasons.^[15]^ Incorporation of the hybrid particles on strip allowed for facile lateral flow assay‐based detection of the three explosives simultaneously down to the lower ppb levels in less than 5 min.

## Results and Discussion

The hybrid gAID systems **MI_1_H_1_A_1_**, **MI_2_H_2_A_2_** and **MI_3_H_3_A_3_** consist of the same mesoporous MCM‐41‐type silica scaffold material (**M**) the pores of which were loaded with three different indicator dyes, 2,7‐dichlorofluorescein (F27=**I_1_**), yielding **MI_1_**, ruthenium tris(bipyridine) (RB3=**I_2_**, for **MI_2_**) and sulforhodamine B (SRB=**I_3_**, for **MI_3_**). Three hapten derivatives, corresponding to the three targeted analytes TATP, TNT and PETN, were then grafted covalently to the outer surface of **MI_1_**‐**MI_3_**, arriving at **MI_1_H_1_**‐**MI_3_H_3_**. Finally, capping of the pores was achieved by binding of the corresponding polyclonal antibodies **A_1_** (anti‐TATP), **A_2_** (anti‐TNT) and **A_3_** (anti‐PETN) to the hapten derivatives, resulting in sensor materials **MI_1_H_1_A_1_**‐**MI_3_H_3_A_3_** (Scheme 1). F27, RB3 and SRB were chosen for two reasons: (i) because the dyes can be excited, and their emission observed, in sufficiently different spectral windows with rather low‐cost optical equipment (Figure S1, Supporting Information) and (ii) because their retention behavior on the strip material is sufficiently different, allowing for chromatographic separation. **A_1_**‐**A_3_** were available in‐house, within BAM′s framework of generating an antibody library against common small‐molecule explosives.^[16]^

Aiming at fast release kinetics in the presence of an analyte yet at efficient pore closure during an analyte's absence, a suitable strategy is to use slightly mismatched hapten derivatives (hapten heterology)^[17]^ and strongly binding antibodies like clones obtained from late immunization boosts during antibody production for the capping biochemistry. In doing so, on rates can be expected to be very high and off rates also still sufficiently fast so that short assay times become possible. This effect is particularly relevant for bivalent antibody binding,^[18]^ which can be expected in this format and has been explained in more detail by us in an earlier publication on a gAID system.^[13a]^ Here, the hapten derivatives **H_1_** to **H_3_** shown in Scheme 1 were employed, which are slightly different from the haptens employed during the immunizations. All the materials prepared were characterized using standard procedures, and the complete preparation and characterization procedures for **MI_1_H_1_A_1_**‐**MI_3_H_3_A_3_** are given in the Supporting Information, Sections 2–5, including Scheme S1, Figures S2–S4 and Table S1.

**Figure 1:**
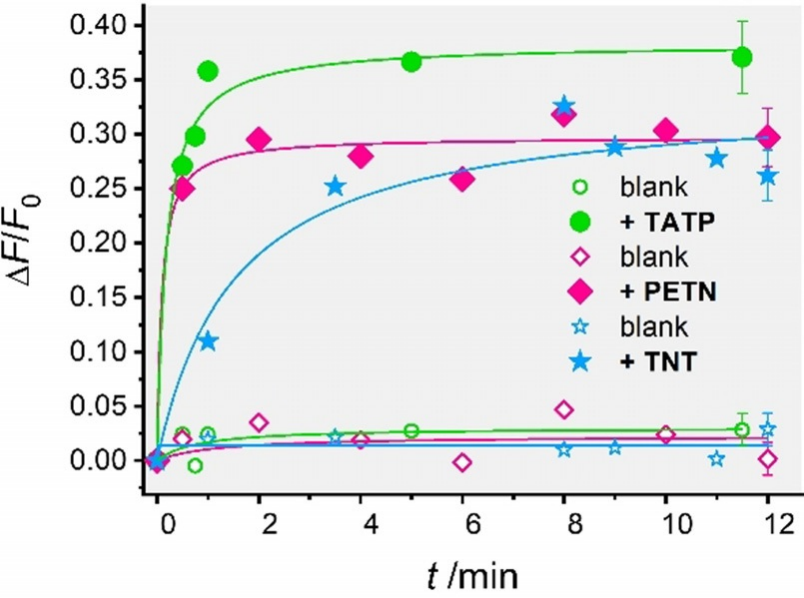
Increase in fluorescence of the three fluorescent dyes F27 (green circles; λ_exc_=490 nm; λ_em_=525 nm), SRB (pink diamonds; λ_exc_=564 nm; λ_em_=588 nm) and RB3 (blue stars; λ_exc_=464 nm; λ_em_=626 nm) vs. time for **MI_1_H_1_A_1_**‐**MI_3_H_3_A_3_** in PBS‐Tween 20 containing 2.5 % MeOH (pH 7.4), in the presence (solid symbols) and the absence (open symbols) of 10.5 ppm TATP, PETN or TNT. The lines are included only as a guide to the eye for better illustration.

The first series of experiments were carried out to assess the individual figures of merit of the responses of **MI_1_H_1_A_1_**‐**MI_3_H_3_A_3_** to their respective analytes. 0.5 mg of **MI_n_H_n_A_n_** were suspended in 4 mL of PBS (phosphate‐buffered saline, 10×) containing Tween 20 (0.05 % v/v) at pH 7.4, yielding suspensions with **MI_n_H_n_A_n_** of 0.12 mg mL^−1^. To study the release kinetics and the effectiveness of pore closure in the absence of the corresponding analyte, i.e., the blank or non‐specific release of the systems, the suspensions were divided into two aliquots. One fraction (2 mL) was spiked with 50 μL of a stock solution of the corresponding analyte in MeOH (450 ppm), equivalent to a final analyte concentration of 10.5 ppm and 2.5 % of MeOH as organic co‐solvent in the suspension. This amount of methanol, which strongly facilitates dosing of the hydrophobic analytes, was tolerated by all antibodies **A_1_**‐**A_3_**. The other fraction was only spiked with the same amount of MeOH, serving as the blank. Fractions of both suspensions (0.25 mL) were then sampled after certain time intervals, centrifuged and the amount of released dye was measured fluorometrically using the appropriate excitation and emission wavelengths for each dye. As can be seen in Figure 1, the presence of PETN and TATP induced a fast opening of the pores with the subsequent release of the entrapped dye. The TNT system was somewhat slower. Control experiments with **MI_1_H_2_A_2_** revealed that this fact was not due to significantly slower uncapping kinetics but was connected to the nature of the loaded indicator (see Figure S5, Supporting Information). Whereas F27 and SRB contained in the materials for TATP and PETN are anionic dyes, the material for TNT contains a cationic dye (RB3). The latter is more strongly retained inside the pores, the walls of which display negatively charged silanol groups.^[19]^ Still, also a >90 % response level was reached in <5 min for **MI_2_H_2_A_2_** when TNT was present in the solution. A beneficial behavior of all three materials is that the blank release in the absence of the corresponding analyte was favorably low (Figure 1).

**Figure 2:**
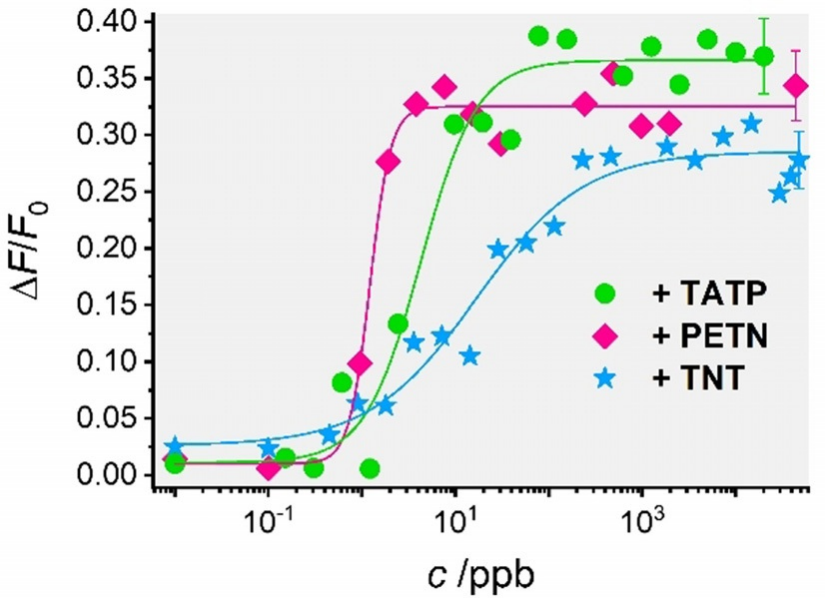
Increase in fluorescence of the three fluorescent dyes SRB (pink diamonds; λ_exc_=564 nm; λ_em_=588 nm), F27 (green circles; λ_exc_=490 nm; λ_em_=525 nm) and RB3 (blue stars; λ_exc_=464 nm; λ_em_=626 nm) vs. concentration of the corresponding analyte for **MI_1_H_1_A_1_**‐**MI_3_H_3_A_3_** in PBS‐Tween 20 containing 2.5 % MeOH (pH 7.4) after 5 min of reaction. The lines exemplify four‐parametric logistic fits.

Following a similar procedure, system sensitivities were assessed by recoding dye release from **MI_n_H_n_A_n_** as a function of the concentration of the different explosives (Figure 2; for spectral response, see Figure S6, Supporting Information). For this purpose, 200 μL of **MI_n_H_n_A_n_** suspensions (0.12 mg mL^−1^ in PBS‐Tween 20 with 2.5 % of MeOH) were stirred for 5 min after the addition of increasing amounts of the explosives. Thereafter, the suspensions were centrifuged, and the fluorescence of the supernatant was measured. A correlation between dye release and analyte concentration was observed, in agreement with the displacement of the antibody from the pore openings due to its interaction with the corresponding explosive. The dose‐response curves were then fitted to a four‐parametric logistic function to derive the limits of detection (LODs),^[20]^ which were determined to 0.3±0.1, 0.4±0.1, and 0.7±0.2 μg L^−1^ for TATP, TNT and PETN, respectively (see Section 12, Supporting Information for details). When explosives were present in the lower ppb range, 2–3 % of the respective dye were released, arriving at signal amplification factors of 60–90 dyes released per antibody displaced.

**Figure 3:**
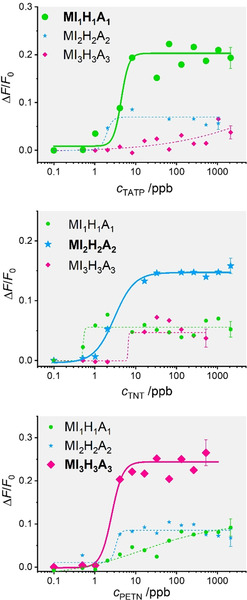
Increase in fluorescence of the three fluorescent dyes SRB (λ_exc_=564 nm; λ_em_=588 nm), F27 (λ_exc_=490 nm; λ_em_=525 nm) and RB3 (λ_exc_=464 nm; λ_em_=626 nm) released from **MI_1_H_1_A_1_**‐**MI_3_H_3_A_3_** in the presence of increasing concentrations of TATP (top), TNT (middle) and PETN (bottom) in PBS‐Tween 20 containing 2.5 % MeOH (pH 7.4) after 5 min of reaction. The lines exemplify four‐parametric logistic fits.

Multiplexing in a single sample is only possible with minimal crosstalk between the different systems, avoiding unmatched (false) positives. We thus performed a triplexed assay using a combination of the three sensing materials in the presence of different concentrations of the three target explosives. Accordingly, suspensions containing mixtures of **MI_1_H_1_A_1_**‐**MI_3_H_3_A_3_** (0.12 mg mL^−1^ from each one in PBS‐Tween 20 containing 2.5 % of MeOH) were left in contact with various amounts of TATP, TNT and PETN as described above. The results in Figure 3 indicate that selective determination is possible. Certain crosstalk was recognized, yet generally, the gAID systems showed a promising selectivity with similar LODs as found previously (1.3±0.6, 0.5±0.1, and 0.7±0.1 μg L^−1^ for TATP, TNT and PETN, respectively). In order to assess the influence of a more demanding matrix, the same experiments were also carried out in milk, and results similar to those found in buffered media were observed, see Supporting Information, Section 8 including Figures S7 and S8.

The selectivity of the capped materials in the presence of other explosives was also studied. Besides TNT, TATP and PETN, the common explosives hexogen (RDX), octogen (HMX), nitroguanidine (NG) and picric acid (PA) were evaluated via similar procedures as described above, using a concentration of the explosives of 2 ppm. As can be seen in Figure S9, Supporting Information, certain cross‐reactivity was found in some cases, yet the selectivity was generally promising for further rapid test development.

**Figure 4:**
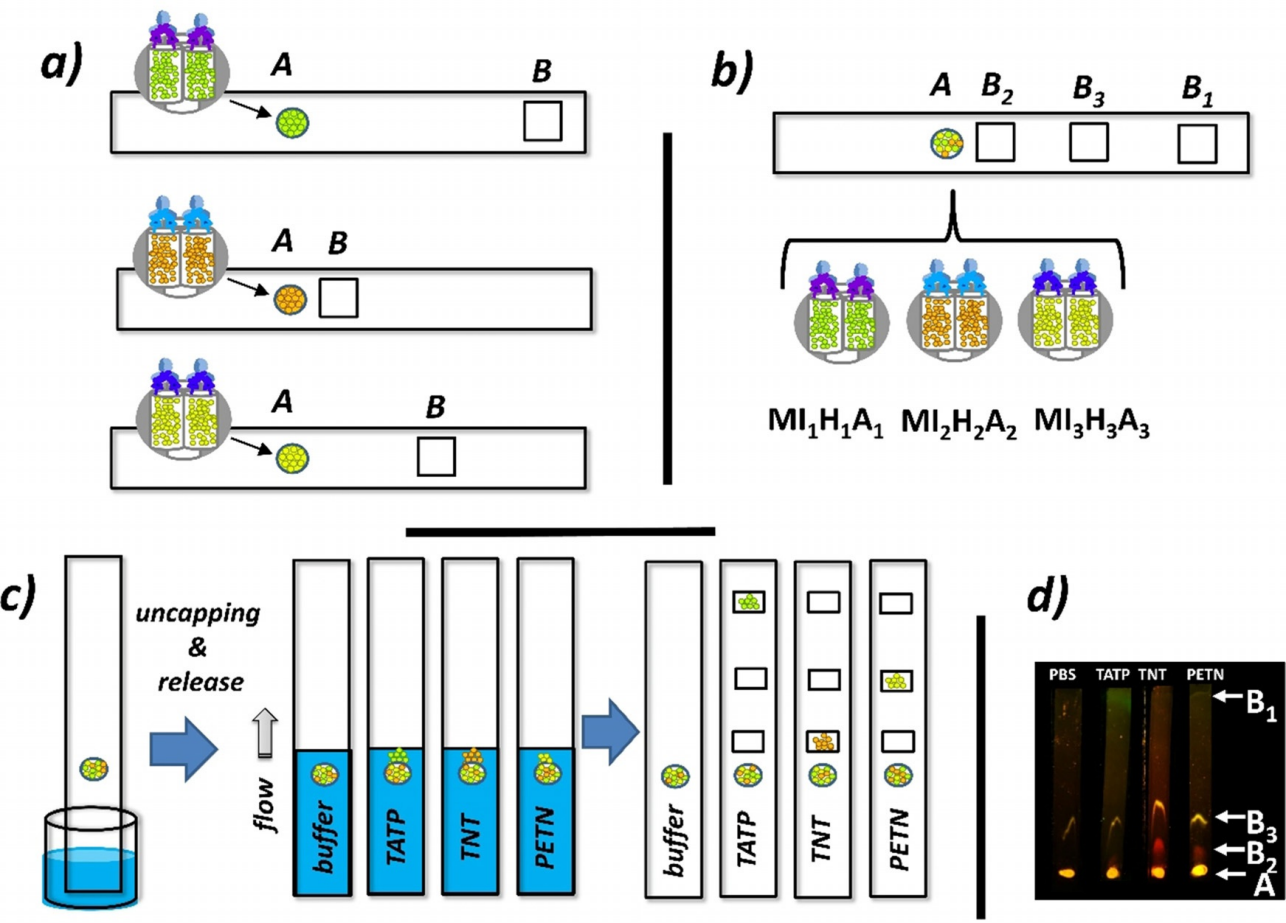
Design and principle of operation of the single‐track multiplexed lateral flow assay. a) Composition of the strips containing **MI_1_H_1_A_1_**, **MI_2_H_2_A_2_**_,_ and **MI_3_H_3_A_3_** independently in zone A. b) Composition of the strips containing a mixture of **MI_1_H_1_A_1_**‐**MI_3_H_3_A_3_** in zone A; note that the labeling of the different zones B_i_ reflects the chromatographic behavior of the dyes. c) Working principle of dye release in the presence of the different explosives TATP, PETN, and TNT. d) Photograph of a set of strips under irradiation with a home‐made light source showing the F27 (**I_1_**), RB3 (**I_2_**) and SRB (**I_3_**) release in the various zones B_1_ of the strips at analyte concentrations of 300 ppb and the blank control (PBS).

Moving towards applicability, the hybrid particles were incorporated with nitrocellulose strips to allow for lateral flow assays (LFAs) with fluorescence readout. The strips contain two different zones, the first zone A, in which the sensing materials are deposited, and a second zone B, which is an area along the strip through which the solvent front travels and in which the signals of the released dyes are collected (Figure 4 a). When the strip is dipped into a solution that does not contain an analyte, no dye is released, and no signal is detected in zone B because the materials remain capped. In the presence of the analyte in the solution, a signal proportional to the analyte concentration is detected in zone B because antibodies are displaced, and dyes are released. The spatial separation of the indicator still contained in closed pores and liberated dyes is based on the fact that the hybrid nanoparticles are large enough to remain at the spot of deposition, whereas the released dye molecules travel with the solvent. The amount of dye released can be quantified with either a fluorescence reader with appropriate excitation and emission wavelengths or a digital camera of a mobile communication device with the respective optical accessories.

First, different strips containing the single materials **MI_1_H_1_A_1_**, **MI_2_H_2_A_2_** and **MI_3_H_3_A_3_** were prepared and tested for their detection capability of TATP, TNT and PETN; similar results as in suspension were obtained (see Supporting Information, Section 10 including Figure S10). In the next step, strips containing mixtures of mass‐equivalent amounts of the three gated materials were prepared and deposited on Hi‐Flow nitrocellulose strips (HF135 from Millipore). The paper support was chosen together with the dyes, to guarantee that F27, RB3 and SRB show sufficiently different retention, allowing the dyes to be separated chromatographically into three different zones B (see Supporting Information, Section 10 including Figure S11). Strips of 0.5×4 cm were cut, and 1 μL of a suspension of a mixture of **MI_1_H_1_A_1_**‐**MI_3_H_3_A_3_** (all at 1 mg mL^−1^) in PBS containing Tween 20 (0.05 % v/v; pH 7.4) were spotted onto zone A (Figure 4 b).

Expecting selective delivery of the dyes in the presence of the corresponding explosives (Figure 4 c), the strips were dipped into buffered solutions (PBS‐Tween 20 with 2.5 % MeOH; pH 7.4) containing TATP, TNT, and PETN at 0.6 ppm. A blank control was also run. After dipping and developing for 4 min, the test strips were dried at room temperature, and the release of the different dyes was measured with a smartphone camera equipped with a 3D‐printed smartphone case fabricated in an analogous way as reported previously by us (see Section 11.1, Supporting Information).^[21]^ Images of the strips were taken with the smartphone under proper light conditions after placing them in the 3D‐printed, customized holder. The intensity of fluorescence of the photographs of the strips was analyzed, extracting the integrated density of fluorescence of the different B_i_ zones with the software ImageJ. Figure S12, Supporting Information shows these results as a function of TATP, TNT and PETN concentrations with the smartphone setup serving as the readout device while Figure S13 depicts the corresponding data obtained with a lateral flow fluorescence reader for comparison (see Supporting information, Section 11). As can be seen, when the sample solution did not contain any explosives, a negligible fluorescence signal was recorded at the different zones B_i_. However, when varying amounts of individual explosives were present, the corresponding release of F27, RB3 or SRB as a function of analyte concentration was observed in zones B_1_‐B_3_ (Figure S12, Supporting Information). Similar sensitivities as in solution were found, arriving at LODs in the lower ppb range (Table 1). Figure 4 d shows a picture under proper light conditions of the corresponding release registered in zones B_1_‐B_3_ with the strips eluted with PBS or a solution of 0.3 ppm of TATP, TNT, or PETN.

**Table 1:** LODs, 5 %‐cut‐off values (X_0.05_) and dynamic ranges for TATP, TNT and PETN from analysis of fluorescence intensities obtained with the reader and the photographs taken under home‐made excitation source.

Devices	TATP			TNT			PETN		
	LOD /ppb	X_0.05_ /ppb	Range /ppb	LOD /ppb	X_0.05_ /ppb	Range /ppb	LOD /ppb	X_0.05_ /ppb	Range /ppb
Reader (single‐track strip)	2.6±0.2	2.9±0.2	10–100	0.7±0.5	0.7±0.5	0.5–50	0.6±0.2	0.6±0.1	1–2
Smartphone (single‐track strip)	0.4±0.1	0.8±0.1	5–300	0.5±0.2	0.5±0.1	2–40	0.8±0.2	1.5±0.2	5–90
Smartphone (multichannel strip)	0.8±0.3	1.2±0.3	5–40	0.6±0.3	0.6±0.3	1–15	0.9±0.3	0.9±0.3	1–30

From the various concentration‐dependent plots shown in this work as well as in studies published by others and us before,[^13c^, ^22^] it is evident that the dynamic response range of these assays is not very broad, commonly spanning between one and two orders of magnitude (see, e.g., the data found for measurement with the smartphone setup in Table 1). This implies that the results of such assays are mainly qualitative to semi‐quantitative. However, as such quick tests are meant to be used directly in the field, in suspect scenarios, the concentration of a target compound can vary tremendously so that commonly a simple “yes/no” response is desired and sufficient to take action, especially when it comes to target compounds such as explosives. Moreover, in a case like explosives detection, when (toxicological) MAC (maximum allowable concentration) values are much less relevant than the threat potential, it is more important to define a so‐called “cut‐off value”, i.e., a value at which the probability for obtaining a false negative response—and thus a trace of explosive escaping detection—is smaller than (commonly) 5 %.^[23]^ The cut‐off values for the present system are also included in Table 1.

In order to investigate the cross‐reactivities against other explosives, several samples containing the explosives RDX, NG, PA and HMX were analyzed with the triple‐material strips. With these explosives, much less fluorescence signal was observed in the different zones, producing a negligible interference (Figure S13d, Supporting Information). The detection of the explosives TATP, TNT and PETN is thus selectively possible by using a single dipstick that contains a mixture of three tailored gAID materials deposited onto one end of the strip.

**Figure 5:**
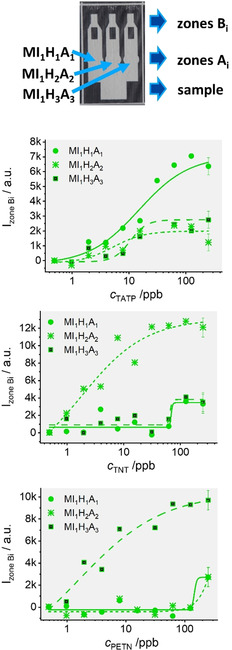
Photograph of a wax‐patterned nitrocellulose strip showing the sample introduction zone, i.e., zones A_i_ containing the corresponding materials **MI_1_H_1_A_1_**, **MI_1_H_2_A_2_**_,_ and **MI_1_H_3_A_3_** and zones B_i_ collecting the released dye in each channel after running the assay (top). Integrated fluorescence density of F27 released and collected in zones B_i_ patterns in the single channels on strips as a function of concentrations of the three different analytes; the fluorescence of a strip ran only with PBS as a blank was subtracted for removal of background fluorescence. The lines exemplify four‐parametric logistic fits.

Despite the promising results found, the single‐track approach presents a limitation if higher‐number multiplexing is desired. We thus combined our hybrid materials with the wax‐patterning of strips, which is a viable alternative to introducing microfluidic features to paper.^[24]^ Multichannel strips with Neptun's fork‐like wax patterning were thus prepared with three separate channels branching out from a single sample reservoir. The features were created on strips of 2.5×1.5 cm with a conventional wax printer. After printing, the strips were heated for 1 min at 110 °C in an oven to guarantee penetration of the wax into the paper, resulting in paper‐thick barriers in the membrane with clear channel separation. The signal readout was accomplished taking pictures of the strips with a smartphone and the differences of the relative luminance in zone B in the presence and in the absence of the corresponding explosives were analyzed to yield the respective data depicted in Figure 5 (see Figure S14, Supporting Information for the corresponding pictures at different concentrations of explosives used).

Table 1 lists the LODs found for the two readout methods and the strips. As can be seen, similar sensitivities were found using the fluorescence reader and the camera. In addition, also, the single‐track and the multichannel approach offered comparable performance. A comparison with data reported in the literature reveals that the performance of the gAID systems is in the range of many ELISAs (enzyme‐linked immunosorbent assays), FIAs (flow immunoassays) and chip‐based immunoassay (IA) systems, which however can always detect only a single analyte, the large majority of the systems having been reported for TNT. Compared with an LFA‐based IA for TNT (entry 26, Table S2, Supporting Information) which, to the best of our knowledge, is the only system that compares to ours in terms of simplicity and portability,^[25]^ the gAID approach is distinctly more sensitive. It can also compete well with three reports on TNT detection with flow cytometry and Luminex® beads, which would allow for multiplexing yet which has not been realized in those works (entries 27–29, Table S2).^[26]^

Although the parallel use of different explosives might not be the most frequent scenario, the simultaneous detection of different species such as mycotoxins, antibiotics, drugs or pesticides would be of paramount importance in the areas of food chemistry, health or environmental monitoring. To elucidate the potential of the present platform for use in such applications, the performance of the strips was also investigated in the presence of mixtures of explosives. For that purpose, several samples containing various mixtures of explosives were measured, also showing favorably low cross‐reactivity (see Supporting Information, Section 11 including Figure S15 for more details).

## Conclusion

In summary, a universal detection system based on antibody‐antigen interaction was developed for the triplexed detection of the explosives TATP, TNT and PETN. The materials prepared allowed for the determination of the explosives within a few minutes with high sensitivity and selectivity, having better performance in comparison with other sensing platforms based on electrochemical^[27]^ or optical detection^[28]^ reported in the literature. The mechanism of the detection relies on a displacement of the antibody from the surface of the mesoporous hybrid material because of highly affine antibody‐explosive interactions, which releases a much larger number of entrapped dye molecules from the pores than antibodies are displaced, entailing a strong amplification of the signal. Because of the modularity of the approach, the concept is easily generalizable and applicable for many small‐molecule analytes. The materials have been incorporated into nitrocellulose strips to approximate a simple dipstick‐based multiplexing assay, allowing for high selectivity and LODs in the lower ppb range. When using a smartphone instead of a reader, the selectivity and sensitivity could be well retained while offering much more flexibility in strip architecture. Because of the versatility of the biomimetic material and the modularity of the assay architecture, it is obvious that this generic approach should be easily transferable to food or environmental analysis, point‐of‐care diagnostics and other areas of application in which the rapid screening for multiple parameters from liquid samples without clean‐up in a dedicated laboratory is in demand. First experiments employing the title materials in milk as a complicated, realistic matrix suggest that such assays might indeed have a broad applicability.

## Conflict of interest

The authors declare no conflict of interest.

## Supporting information

As a service to our authors and readers, this journal provides supporting information supplied by the authors. Such materials are peer reviewed and may be re‐organized for online delivery, but are not copy‐edited or typeset. Technical support issues arising from supporting information (other than missing files) should be addressed to the authors.

Supplementary
