# Supplementary material for: Multiplexed Detection of Analytes on Single Test Strips with Antibody‐Gated Indicator‐Releasing Mesoporous Nanoparticles
Source: Angew Chem Int Ed Engl. 2020 Oct 1;59(52):23862–9. doi: 10.1002/anie.202009000 (PMC7756650; doi:10.1002/anie.202009000)
Supplement: Supplementary file 1 — Supplementary [file ANIE-59-23862-s001.pdf]

Angewandte Chemie
Eine Zeitschrift der Gesellschaft Deutscher Chemiker

Supporting Information

Multiplexed Detection of Analytes on Single Test Strips with Antibody-Gated Indicator-Releasing Mesoporous Nanoparticles

*Estela Climent, Mustafa Biyikal, Delia Gröninger, Michael G. Weller, Ramón Martínez-Máñez, and Knut Rurack**

anie_202009000_sm_miscellaneous_information.pdf

SUPPORTING INFORMATION

Table of Contents

TABLE OF CONTENTS 2
EXPERIMENTAL PROCEDURES 3
1 GENERAL METHODS 3
2 SYNTHESIS OF HAPTENS AND HAPTEN DERIVATIVES 3
3 POLYCLONAL ANTIBODIES FOR TATP, TNT AND PETN 4
4 SYNTHESIS AND CHEMICAL CHARACTERIZATION OF MATERIALS 5
4.1 SYNTHESIS OF MCM-41 MESOPOROUS SILICA NANOPARTICLE SUPPORT (MCM) 5
4.2 SYNTHESIS OF MI_1H_1 5
4.3 SYNTHESIS OF MI_2H_2 5
4.4 SYNTHESIS OF MI_3H_3 5
4.5 SYNTHESIS OF MI_1H_2 AND MI_1H_3 5
4.6 SYNTHESIS OF ANTIBODY-CAPPED MATERIALS $MI_NH_NA_N$ 5
5 CHARACTERIZATION OF MATERIALS 5
RESULTS AND DISCUSSION 9
6 CONTROL EXPERIMENTS WITH $MI_1H_2A_2$ 9
7 RAW SPECTRA OF RESPONSE TO ANALYTES 9
8 DETECTION OF EXPLOSIVES IN TURBID MEDIA: MILK AS AN EXAMPLE 9
9 CROSS-REACTIVITIES 10
10 LATERAL FLOW TESTS WITH FLUORESCENCE READ-OUT PROTOCOL 11
11 MULTIPLEXING DETECTION OF EXPLOSIVES TATP, TNT AND PETN 12
11.1 SMARTPHONE SETUP AS READOUT DEVICE WITH A SINGLE STRIP 12
11.2 FLUORESCENCE READER AS READOUT DEVICE WITH A SINGLE STRIP 12
11.3 SMARTPHONE SETUP AS READOUT DEVICE WITH PATTERNED STRIPS 13
12 ADDITIONAL NOTES ON ANALYTICAL PERFORMANCE 15
12.1 LIMITS OF DETECTION 15
12.2 REPEATABILITY 16
12.3 STABILITY 16
12.4 CUT-OFF VALUES 16
12.5 PREVIOUSLY REPORTED IMMUNOASSAYS FOR EXPLOSIVES DETECTION 16
REFERENCES 18
AUTHOR CONTRIBUTIONS 18

Experimental Procedures

1 General methods

Chemicals and solvents were purchased from Sigma-Aldrich, ACBR, Merck and J.T. Baker. Buffers were prepared with ultrapure reagent water, which was obtained by running demineralized water (by ion exchange) through a Milli-Q® ultrapure water purification system (Millipore Synthesis A10). Phosphate-buffered saline (PBS 10X; 70 mmol dm^{-3} Na_2HPO_4, 10 mmol dm^{-3} NaH_2PO_4, 145 mmol dm^{-3} NaCl, pH 7.6), PBS containing 0.005% BSA (w/v), 0.05% Tween-20 (v/v) and 200 ppm of the corresponding dye were used for capping processes. Controlled release experiments were performed using a solution of PBS 10X containing 0.05% Tween-20 (v/v) and 2.5% EtOH. The common explosives PETN, TNT, RDX and HMX were obtained from BAM's *Testing and Evaluation of Explosives and Pyrotechnics Division*, and nitroguanidine (containing ca. 25% water) were purchased from Sigma Aldrich.

Absorption and fluorescence spectroscopy, elemental analysis, thermogravimetric analysis (TGA), transmission electron microscopy (TEM), N_2 adsorption-desorption, dynamic light scattering (DLS), mass spectrometry and NMR techniques were employed to characterize the synthesized materials and test their behaviour towards the corresponding analytes. UV-vis spectra were measured with a Specord 210plus from Analytik Jena. Fluorescence measurements were carried out on a Fluoromax4 from HORIBA Scientific. Thermogravimetric analyses were carried out on a STA7200 (Hitachi High-Tech Analytical Science) thermobalance, using in a first step a nitrogen atmosphere (80 mL min^{-1}) with a heating program consisting of a ramp of 10 °C min^{-1} from 25 °C to 600 °C and in a second step an oxidizing atmosphere (air, 80 mL min^{-1}) from 600 °C until 1000 °C with a heating program consisting of a ramp of 10 °C min^{-1}. Elemental analyses were carried out with a Euro EA-Elementaranalysator. TEM images were obtained with a Tecnai G2 20 Twin Transmission Electron Microscope, FEI Company. N_2 adsorption-desorption isotherms were recorded with a Micromeritics ASAP2010 automated sorption analyzer. The samples were degassed for 3h at 200 °C in a vacuum. The specific surface areas were calculated from the adsorption data in the low-pressure range using the Brunauer-Emmett-Teller (BET) model. Pore sizes were determined following the Barrett-Joyner-Halenda (BJH) method. DLS studies were conducted using a Malvern Zetasizer Nano ZS. Mass spectra were measured with a CT Premier XE-TOF mass spectrometer. ^1H and ^{13}C NMR spectra were acquired with Bruker AV-500 and AV-600 spectrometers, using residual protonated solvents as internal standards (^1H: $\delta[CDCl_3] = 7.24$ ppm and ^{13}C: $\delta[CDCl_3] = 77.23$ ppm). Fluorescence measurements on strips were recorded with an ESE-Quant FL and a universal strip holder from Qiagen. For the smartphone-based approach, a 3D-box was printed with black PLA using an Ultimaker 3 printer. LEDs and optical filters were purchased from Thorlabs. Photographs were taken with a Samsung Galaxy S7 and values retrieved from images via the integrated density with the software ImageJ, i.e., the product of mean grey value $\bar{G}$, $\bar{G}$ = (red + green + blue)/3, and the selected area *a* (in square pixels)

2 Synthesis of haptens and hapten derivatives

TATP, TNT and PETN hapten derivatives $\mathbf{H_1}$, $\mathbf{H_2}$ and $\mathbf{H_3}$ employed to prepare the corresponding sensing materials were prepared through an amidation reaction of the corresponding haptens **I**, **II** or **III** with (3-aminopropyl)triethoxysilane (APTES) using a modification of the active ester method (see Scheme S1).[1] Haptens **I**, **II** and **III** were prepared following previously described procedures.[2] For the hapten derivatives, three different solutions, each containing *N*-hydroxysuccinimide (NHS, 18.89 mg, 0.16 mmol) and *N,N'*-dicyclohexylcarbodiimide (DCC, 33.86 mg, 0.16 mmol) dissolved in anhydrous THF (0.75 mL), were prepared. Then, the individual solutions were added to a solution of hapten **I**, **II** or **III** (0.14 mmol) in anhydrous THF (0.25 mL). The mixtures were stirred at room temperature for 5 h, and the white solids formed (dicyclohexylurea) were removed from each fraction by centrifugation. In a second step, three portions of APTES (33.53 µL, 0.14 mmol) were added to each solution and the reaction mixtures were stirred for 24 h at room temperature. The remaining dicyclohexylurea of the different reactions was removed by centrifugation and the solvent was evaporated from each fraction under reduced pressure to give yellow sticky oils $\mathbf{H_1}$, $\mathbf{H_2}$ and $\mathbf{H_3}$ (0.12 mmol, 0.13 mmol and 0.12 mmol; yields 85.7 %, 92.8% and 85.7%), respectively.

TATP-based $\mathbf{H_1}$: ^1H NMR ($CDCl_3$): δ (ppm) = 0.63 (t, 2 H, $-CH_2-Si-$), 0.96 (q, 6H, CH_3CH_2-C-) 1.22 (t, 9 H, $SiO-CH_2-CH_3$) 1.22 (m, 2H, $-NH-CH_2-CH_2-CH_2-Si-$), 1.32 (s, 9H, CH_3-C-) 1.58 (m, 8H, $CH_2-CH_2-CH_2-CH_2-CH_2-CO-NH-$) 1.84 (m, 4 H, CH_3CH_2-C-), 2.16 (m, 2 H, $-CH_2-CH_2-CH_2-CH_2-CH_2-CO-NH-$), 3.24 (t, 2H, $-NH-CH_2-CH_2-CH_2-Si-$), 3.81 (s, 6H, CH_3-CH_2-O-Si). ^{13}C NMR ($CDCl_3$): 7.7, 18.3, 18.6, 21.3, 22.8, 23.7, 24.9, 25.3, 25.6, 29.4, 33.8, 36.8, 41.8, 58.5, 107.4, 109.3, 173.1. Exact mass (M+H) 554.336; found 554.326.

TNT-based $\mathbf{H_2}$: ^1H NMR ($CDCl_3$): ($CDCl_3$): δ (ppm)= 0.65 (t, 2 H, $-CH_2-Si-(O-CH_2-CH_3)_3$), 1.22 (t, 9H, $-CH_2-Si-(O-CH_2-CH_3)_3$), 1.62 (m, 6H, $-CH_2-CH_2-CH_2-CH_2-CO{=}NH-CH_2-CH_2-$), 2.16 (m, 2H, $-CH_2-CH_2-CH_2-CO{=}NH-$), 2.97 (t, 2H, $-CH_2-CO{=}NH-$), 3.25 (t, 2H, $-CH_2-CH_2-CH_2-CH_2-CH_2-CO{=}NH-$), 3.48 (t, 2H, $-CH_2-CO{=}NH-CH_2-$), 3.82 (q, 6H, $Si-(O-CH_2-CH_3)_3$), 7.57 (d, 1H, $C_6H_3(NO_2)_2$), 8.35 (d, 1H, $C_6H_3(NO_2)_2$), 8.73 (s, 1H, $C_6H_3(NO_2)_2$). ^{13}C NMR ($CDCl_3$): 7.7, 18.3, 22.8, 25.4, 25.6, 28.3, 32.8, 36.4, 41.8, 58.4, 120.2, 124.2, 126.8, 133.2, 144.5, 169.1. Exact mass (M+H) 486.2193; found 486.2278.

PETN-based $\mathbf{H_3}$: ^1H-NMR ($CDCl_3$) δ (ppm): 0.63 (t, 2 H, $-CH_2-Si-(O-CH_2-CH_3)_3$), 1.22 (t, 9H, $-CH_2-Si-(O-CH_2-CH_3)_3$), 1.30 (m, 2H, $-CO-O-CH_2-CH_2-CH_2-CH_2-CH_2-CO-NH-$), 1.60 (m, 4H, $-CO-O-CH_2-CH_2-CH_2-CH_2-CH_2-CO-NH-$), 1.90 (m, 2H, $-NH-CH_2-CH_2-CH_2-Si-$), 2.16 (t, 2H, $-CO-O-CH_2-CH_2-CH_2-CH_2-CH_2-CO-NH-$), 3.24 (t, 2H, $-NH-CH_2-CH_2-CH_2-Si-$), 3.81 (q, 6H, $Si-(O-CH_2-CH_3)_3$), 4.09 (t, 2H,

-C- CH_2-O-CO-O), 4.21 (s, 2H, -CO-O-CH_2-CH_2-CH_2-CH_2-CH_2-CO-NH-), 4.68 (s, 6H, NO_2- CH_2-C-). ^{13}C NMR ($CDCl_3$): 7.7, 18.3, 22.8, 25.4, 25.6, 28.3, 36.4, 41.8, 49.1, 58.4, 64.5, 67.1, 69.5, 154.2, 172.4. Exact mass (M+H) 633.2209; found 633.2294.

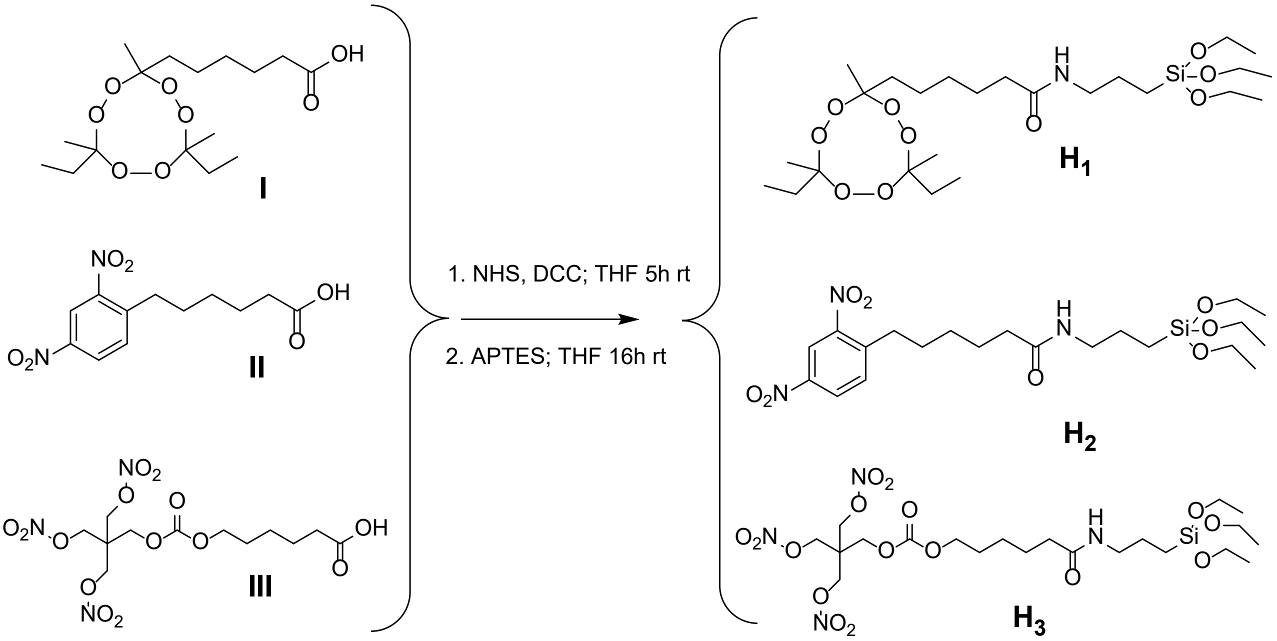

Scheme S1. Synthesis of TATP, TNT and PETN hapten derivatives **H_1**, **H_2** and **H_3** from haptens **I**, **II** and **III**.

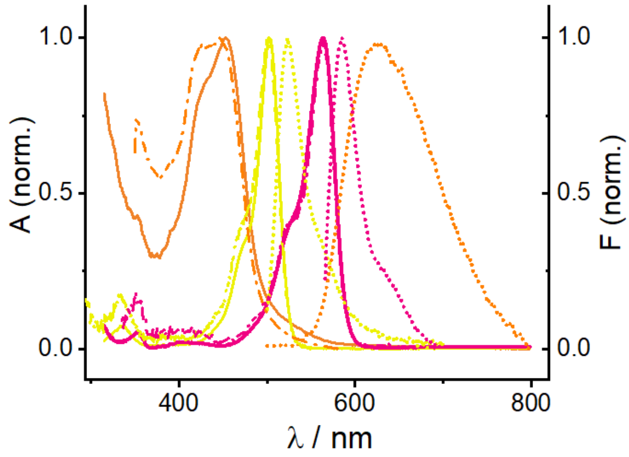

Figure S1. Absorbance (solid), fluorescence excitation (dash-dotted) and emission spectra (dotted lines) of the dyes F27, SRB and RB3 in PBS (80 mM) containing 2.5% MeOH.

3 Polyclonal antibodies for TATP, TNT and PETN

The polyclonal antibodies against TATP, TNT and PETN were obtained as described previously by us.[2a-c] For immunizing purposes, the corresponding haptens were covalently attached, through their carboxylic acid moieties to the lysine residues of BSA, using a modification of the active-ester method. The conjugates were employed to immunize six 9 to 12 weeks old (2–2.5 kg) rabbits with sub-cutaneous injections (two rabbits per analyte), and 8–12 different sera were obtained from each rabbit. These sera were used to evaluate the titer and affinity maturation of the hapten-specific antibodies via ELISA. Based on these results, the sera of boosts 11, 8 and 5 for TATP, TNT and PETN were selected as the best suited for the preparation of the antibody-capped hybrid materials.

4 Synthesis and chemical characterization of materials

4.1 Synthesis of MCM-41 mesoporous silica nanoparticle support (MCM)

Mesoporous MCM-41 nanoparticles were synthesized as reported previously.[3] *n*-Cetyltrimethylammonium bromide (CTABr, 1.00 g, 2.74 mmol) was first dissolved in 480 mL of deionized water. Then, 3.5 mL of 2 M NaOH in deionized water were added to the CTABr solution, followed by heating the solution to 80 ºC. Tetraethylorthosilicate (TEOS) (5.00 mL, 2.57×10^{-2} mol) was then added dropwise to the surfactant solution. The mixture was stirred for 2 h at 80°C to give a white precipitate. Finally, the solid product was centrifuged, washed with deionized water and ethanol, and dried at 60ºC (MCM-41 as-synthesized). Removal of the template was achieved by calcination for 8 h at 550°C, yielding the calcinated porous material **M**.

4.2 Synthesis of $\mathbf{MI_1H_1}$

MCM-41 mesoporous nanoparticles **M** (100 mg) and the dye 2,7-dichlorofluorescein sodium salt (F27 or $\mathbf{I_1}$; 33.0mg, 0.8 mmol g^{-1} solid) were suspended in EtOH (5 mL). The suspension was stirred for 24 h at rt to achieve maximum loading of the dyes into the pores of the MCM-41 scaffold. Afterwards, 25 µmol of $\mathbf{H_1}$ were dissolved in 40 µL THF and the final mixture was stirred for 6 h at room temperature. Finally, the solid $\mathbf{MI_1H_1}$ was centrifuged (5 min at 9500 rpm), washed with EtOH (1 mL) and isolated by centrifugation, before drying for 12 h at 35 ºC in a vacuum.

4.3 Synthesis of $\mathbf{MI_2H_2}$

$\mathbf{MI_2H_2}$ was prepared following the same procedure as for $\mathbf{MI_1H_1}$, i.e., **M** (100 mg) and the dye tris(bipyridine)ruthenium(II) chloride (RB3 or $\mathbf{I_2}$; 59 mg, 0.8 mmol g^{-1} solid) were suspended in acetonitrile (10 mL), stirring the suspension for 24 h at rt. Afterwards, 12.5 µmol of $\mathbf{H_2}$ were dissolved in 40 µL THF and the final mixture was stirred for 6 h at room temperature. Finally, the solid $\mathbf{MI_2H_2}$ was centrifuged (5 min at 9500 rpm), washed with acetonitrile (1 mL) and isolated by centrifugation, before drying for 12 h at 35 ºC in a vacuum.

4.4 Synthesis of $\mathbf{MI_3H_3}$

$\mathbf{MI_3H_3}$ was prepared following the same procedure as for $\mathbf{MI_1H_1}$, i.e., **M** (100 mg) and the dye sulforhodamine B sodium salt (SRB or $\mathbf{I_3}$; 46.5 mg, 0.8 mmol g^{-1} solid) were suspended in acetonitrile (20 mL), stirring the suspension for 24 h at rt. Afterwards, 12.5 µmol of $\mathbf{H_3}$ were dissolved in 40 µL THF and the final mixture was stirred for 6 h at room temperature. Finally, the solid $\mathbf{MI_3H_3}$ was centrifuged (5 min at 9500 rpm), washed with acetonitrile (1 mL) and isolated by centrifugation, before drying for 12 h at 35 ºC in a vacuum.

4.5 Synthesis of $\mathbf{MI_1H_2}$ *and* $\mathbf{MI_1H_3}$

Materials $\mathbf{MI_1H_2}$ and $\mathbf{MI_1H_3}$, both containing F27 = $\mathbf{I_1}$ as an indicator in the pores yet also the other two hapten derivatives $\mathbf{H_2}$ and $\mathbf{H_3}$ grafted to the surface, were analogously prepared by suspending **M** (100 mg) and $\mathbf{I_1}$ (33.0mg, 0.8 mmol g^{-1} solid) in EtOH (5 mL). After 24 h stirring at rt, the suspension was divided into two fractions, and 25 µmol of $\mathbf{H_2}$ and $\mathbf{H_3}$, dissolved in 15 µL THF, were added to each fraction. The final mixtures were stirred for 6 h at room temperature. Finally, the solids $\mathbf{MI_1H_2}$ and $\mathbf{MI_1H_3}$ were centrifuged (5 min at 9500 rpm), washed with EtOH (1 mL) and isolated by centrifugation before drying for 12 h at 35 ºC in a vacuum.

4.6 Synthesis of antibody-capped materials $\mathbf{MI_nH_nA_n}$

$\mathbf{MI_1H_1A_1}$, $\mathbf{MI_2H_2A_2}$, $\mathbf{MI_3H_3A_3}$, $\mathbf{MI_1H_2A_2}$ and $\mathbf{MI_1H_3A_3}$ were prepared using a modified procedure reported previously.[2d] 1 mg of the corresponding materials $\mathbf{MI_1H_1}$, $\mathbf{MI_2H_2}$, $\mathbf{MI_3H_3}$, $\mathbf{MI_1H_2}$ and $\mathbf{MI_1H_3}$ were suspended in 0.5 mL of a solution of phosphate-buffered saline (PBS) at pH 7.4 containing 200 ppm of the respective dye, the respective antibody in PBS (4.5 µL A1-3 mg S1-3-1), 0.005% (w/v) bovine serum albumin (BSA) and 0.05% (v/v) Tween-20 and stirred for 20 h at room temperature. Afterwards, the capped materials $\mathbf{MI_1H_1A_1}$, $\mathbf{MI_2H_2A_2}$, $\mathbf{MI_3H_3A_3}$, $\mathbf{MI_1H_2A_2}$ and $\mathbf{MI_1H_3A_3}$ were isolated by centrifugation and washed several times with PBS, before drying in a vacuum as reported above. All the capped materials were stored in a dry state at 4°C.

5 Characterization of materials

All solids prepared were characterized using standard procedures. The presence of the mesoporous structure was confirmed with nitrogen adsorption-desorption isotherms and transmission electron microscopy (TEM) analysis, whereas the respective contents of

antibody, hapten and dye in and on the materials were estimated from elemental analysis, thermogravimetry and absorbance measurements. TEM images revealed that the as-prepared MCM-41 nanoparticles **M** are obtained as spherical particles with diameters ranging of 70–110 nm. The micrographs also show the hexagonal mesoporosity and the typical channels of the MCM-41 matrix, visualized as alternating black and white stripes (Figures S2a, S2b). Figure S2b also shows a TEM image of the capped material **MI$_1$H$_1$A$_1$**. Here, it is possible to observe an increase in the disorder of the mesochannels, ascribed to the increase of organic matter (antibodies and other proteins) on the surface.

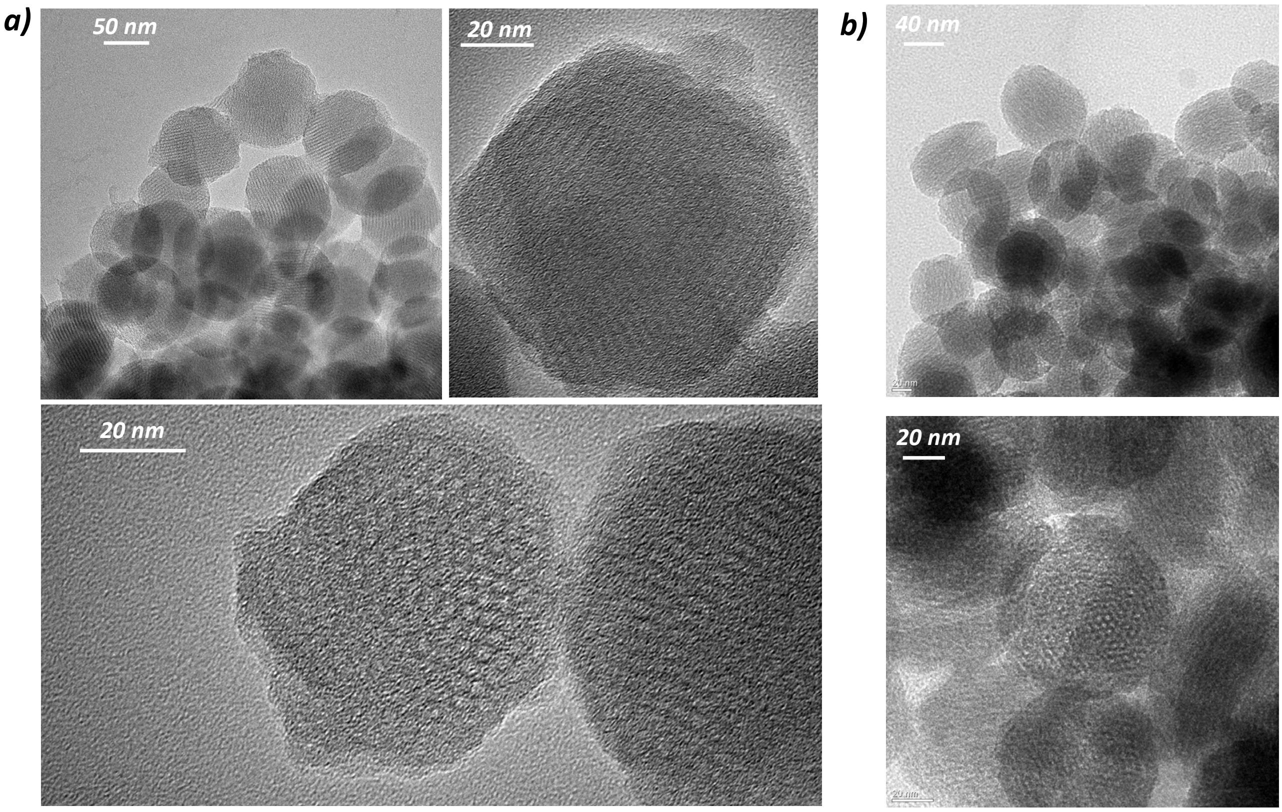

Figure S2. TEM micrographs of a) MCM-41 nanoparticles **M** and b) capped material **MI$_1$H$_1$A$_1$,** showing the typical hexagonal porosity of the MCM-41 mesoporous matrix and the change of appearance on the surface on the MCM-41 support after the various steps of functionalization.

Nitrogen adsorption-desorption isotherms of the calcinated MCM-41 nanoparticles are shown in Figure S3. A behaviour typical for mesoporous solids can be recognized, consisting of an adsorption step at intermediate P/P_0 values (0.25–0.35). This curve corresponds to a type-IV isotherm, in which the observed step is characteristic for nitrogen condensation inside the mesopores. No hysteresis loop is observed in this interval which, together with the narrow BJH pore distribution, is indicative of uniform cylindrical mesopores. Employing the BJH model on the adsorption branch of the isotherm, the pore diameter can be determined to 2.15 nm and the pore volume to 0.85 cm^3 g^{-1}. Furthermore, the application of the BET model resulted in a value of 999.3 m^2 g^{-1} for the total specific surface area. A second adsorption step at a higher relative pressure (P/P_0>0.9) is also observed, which corresponds to the filling of the large voids between the particles (pore volume of 0.55 cm^3 g^{-1} calculated according to the BJH model) and can, therefore, be ascribed to textural porosity. N_2 adsorption-desorption isotherms of the functionalized materials could not be obtained because of the large amount of material that would have been to be prepared. However, significantly reduced pore volumes and specific surface areas would be expected already for dye-loaded materials **MI$_1$**, **MI$_2$** and **MI$_3$**.

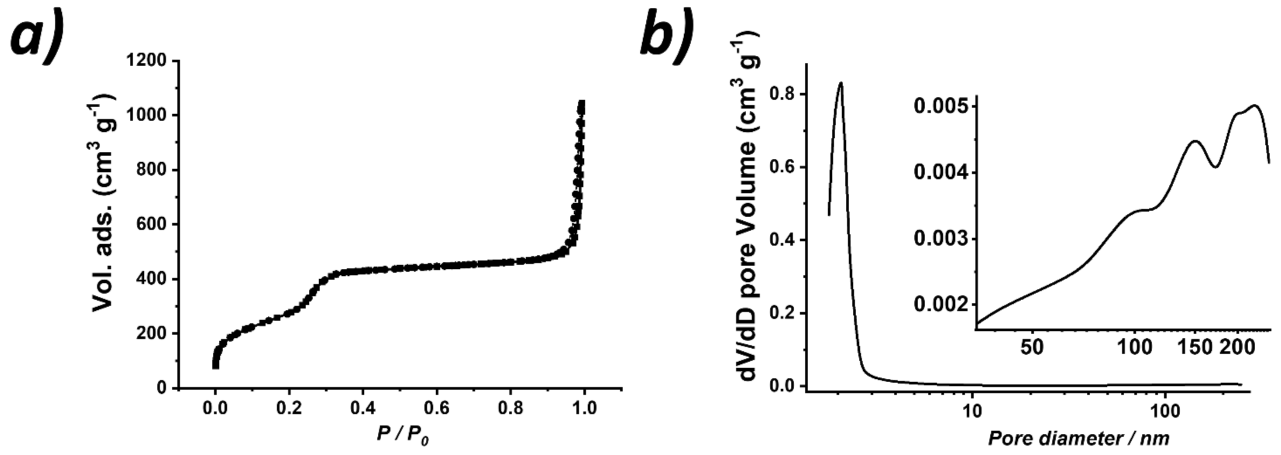

Figure S3. a) N_2 adsorption-desorption isotherm for calcined MCM-41 nanoparticles **M**. b) Pore size distribution of **M**. Inset shows a zoom out into the 50–200 nm region of the pore size distribution, ascribed to the textural porosity.

The size of the particles was also determined by dynamic light scattering (DLS). All measurements were performed in triplicate on previously sonicated and highly diluted Milli-Q water dispersions (100 µg mL^{-1}) at pH 6. The results of the measurements of **M**, **MI$_2$H$_2$** and **MI$_2$H$_2$A$_2$** are shown in Figure S4 as representative examples, revealing mean hydrodynamic diameters of 122 ± 25, 110 ± 15 and 205 ± 35 nm for **M**, **MI$_2$H$_2$** and **MI$_2$H$_2$A$_2$**, respectively. The differences in the particle diameters between **M** and **MI$_2$H$_2$** are minor, but the increase upon antibody attachment is significant, which is because, along with the antibodies, stabilizing BSA is loosely attached as well during the incubation step used for capping.[4]

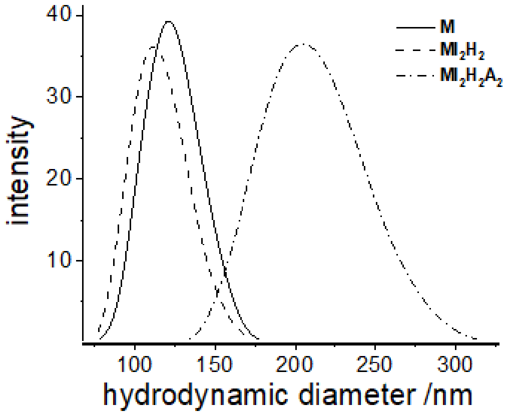

Figure S4. Hydrodynamic diameter measured by DLS for **M**, **MI$_2$H$_2$** and **MI$_2$H$_2$A$_2$**.

The contents of hapten derivatives **H$_1$**, **H$_2$** and **H$_3$** as well as dyes F27, RB3 and SRB in the prepared solids were determined by elemental analysis and thermogravimetric studies. Furthermore, the amounts of loaded dye in the final systems after antibody capping were determined indirectly from the difference between the amount measured for **MI$_n$H$_n$** and the amount washed out into the aqueous washing fractions after the capping process.

Table S1. Contents (in µmol g^{-1} solid) of the hapten derivatives **H_1**, **H_2** and **H_3** and the dyes F27, RB3 and SRB in the prepared solids.

Solid	TATP hapten **H_1**	TNT hapten **H_2**	PETN hapten **H_3**	F27 (**I_1**)	RB3 (**I_2**)	SRB (**I_3**)
**MI_1H_1**	111.8	–	–	213.4	–	–
**$MI_1H_1A_1$**	111.8	–	–	190.4	–	–
**MI_2H_2**	–	65.7	–	–	310.0	–
**$MI_2H_2A_2$**	–	65.7	–	–	124.8	–
**MI_3H_3**	–	–	33.5	–	–	232.5
**$MI_3H_3A_3$**	–	–	33.5	–	–	140.9
**MI_1H_2**	–	98.1	–	172.2	–	–
**$MI_1H_2A_2$**	–	98.1	–	143.0	–	–
**MI_1H_3**	–	–	85,1	156.5	–	–
**$MI_1H_3A_3$**	–	–	85,1	127.3	–	–

Results and Discussion

6 Control experiments with $MI_1H_2A_2$

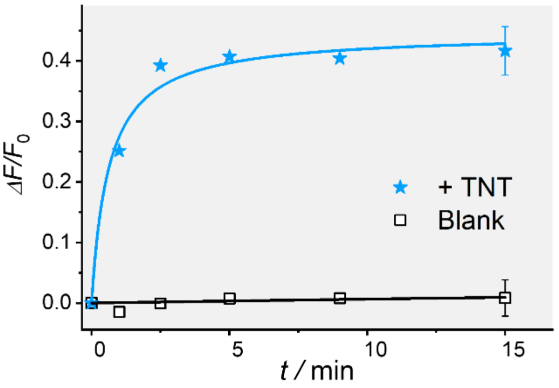

Figure S5. Increase in fluorescence vs time for F27 (λ_{exc} = 490 nm; λ_{em} = 525 nm) released from **$MI_1H_2A_2$** in PBS-Tween 20 containing 2.5% MeOH (pH 7.4), in the presence (circles) and the absence (stars) of 10.5 ppm of TNT. The lines are included only as a guide to the eye for better illustration.

7 Raw spectra of response to analytes

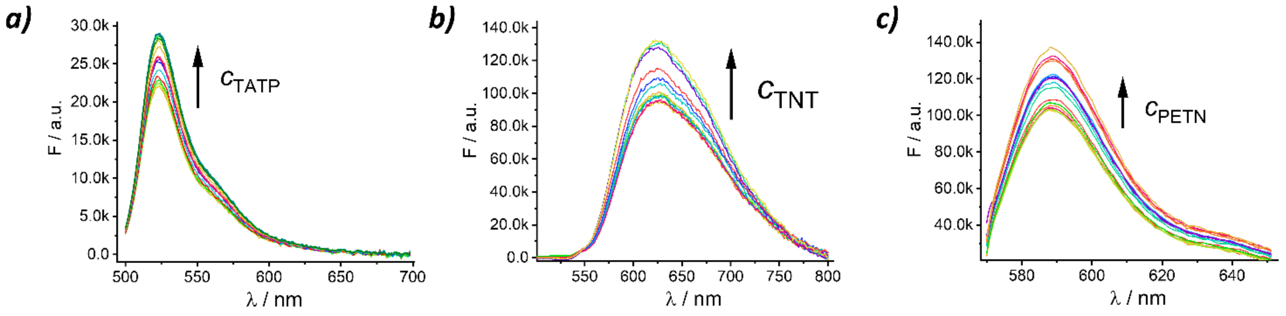

Figure S6. Increase in fluorescence of the three fluorescent dyes a) F27 (λ_{exc} = 490 nm), b) RB3 (λ_{exc} = 460 nm) and c) SRB (λ_{exc} = 560 nm) vs concentration of the corresponding analyte for **$MI_1H_1A_1$–$MI_3H_3A_3$** in PBS-Tween 20 containing 2.5% MeOH (pH 7.4) after 5 min of reaction.

8 Detection of explosives in turbid media: milk as an example

Rapid tests for point-of-need measurements have to be applicable in real samples without the possibility of extensive clean-up. To assess the general suitability of the title materials, the suspensions assays were thus also performed in milk containing 3.5 % fat. Milk was chosen because it is a complicated liquid sample that (i) is turbid so that it cannot usually be used directly in spectroscopic analysis, (ii) it is a liquid food stuff, food stuff being one of the sample types frequently encountered in explosives-related security applications and (iii) it contains a high load of a variety of matrix components, in so far also being related to soil extracts which would be typical sample types in environmental analysis. As can be seen in Figure S7, the detection of TATP, TNT and PETN using materials **$MI_1H_1A_1$, $MI_2H_2A_2$** and **$MI_3H_3A_3$** could also be successfully performed and results similar to those in buffered media were found.

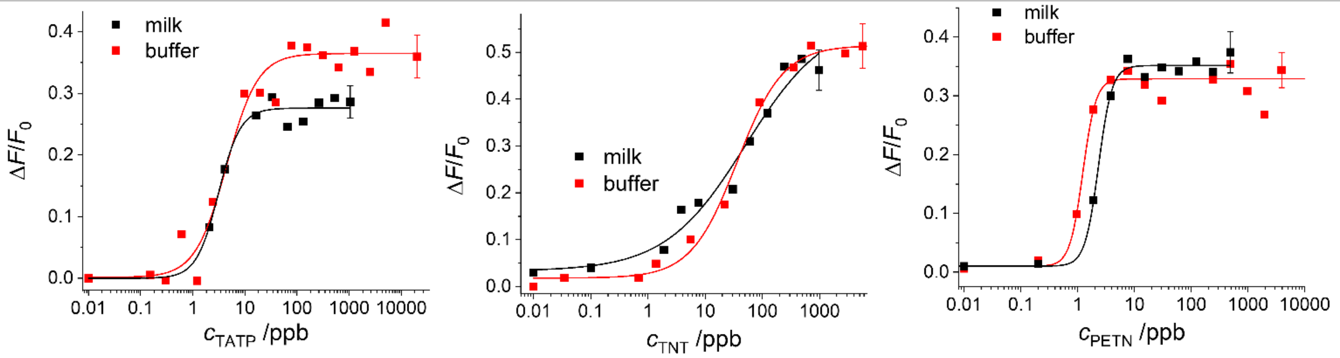

Figure S7. Increase in fluorescence of, from left to right, F27 (λ_{exc} = 490 nm; λ_{em} = 525 nm), RB3 (λ_{exc} = 464 nm; λ_{em} = 626 nm) and SRB (λ_{exc} = 564 nm; λ_{em} = 588 nm) released from **$MI_1H_1A_1$** (left), **$MI_2H_2A_2$** (middle) and **$MI_3H_3A_3$** (right) in the presence of increasing concentrations of TATP (left), TNT (middle) and PETN (right) in full-fat milk (3.5 % fat, black) and PBS-Tween 20 containing 2.5% MeOH (pH 7.4, red) after 5 min of reaction. The lines exemplify four-parametric logistic fits.

Following a similar approach, the crosstalk within the three systems was also tested using a cocktail of all three materials. The response in milk was again found to be comparable to that in buffered media, Figure S8.

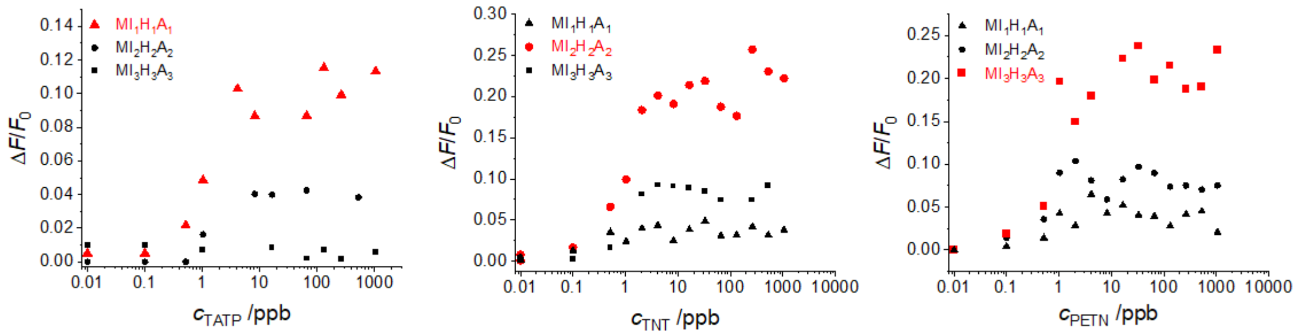

Figure S8. Increase in fluorescence of F27 = **I_1** (λexc = 490 nm; λem = 525 nm), RB3 = **I_2** (λexc = 464 nm; λem = 626 nm) and SRB = **I_3** (λexc = 564 nm; λem = 588 nm), released from **$MI_1H_1A_1$–$MI_3H_3A_3$** in the presence of increasing concentrations of TATP (left), TNT (middle) and PETN (right) in full-fat milk (3.5 % fat) after 5 min of reaction).

9 Cross-reactivities

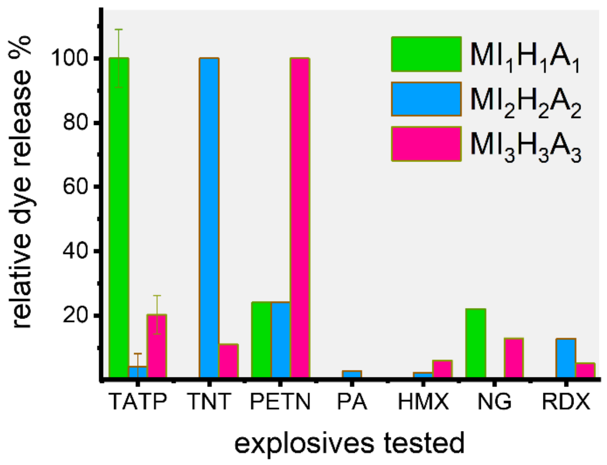

Figure S9. Relative dye release of the three sensory materials **$MI_1H_1A_1$, $MI_2H_2A_2$** and **$MI_3H_3A_3$** in the presence of the target analytes, the primary competitors of the tri-plexing assay and other common explosives such as picric acid (PA), octogen (HMX), nitroguanidine (NG) and hexogen (RDX).

10 Lateral flow tests with fluorescence read-out protocol

Nitrocellulose strips of 0.5 × 4 cm were prepared, and 1 µL of a suspension (1 mg mL^{-1}) of the corresponding materials **MI$_n$H$_n$A$_n$** was deposited at ca. 1 cm from one end of the strip (zone A). The strips containing materials **MI$_1$H$_1$A$_1$**, **MI$_2$H$_2$A$_2$** or **MI$_3$H$_3$A$_3$** were then dipped into the PBS-buffered sample solutions containing different concentrations of the respective analytes TATP, TNT and PETN, and the flow was left to develop for 3 min. Afterwards, the strips were dried at room temperature and the fluorescence was measured using a flow assay reader at 520 nm (λ_{exc} = 470 nm) for **MI$_1$H$_1$A$_1$**, 625 nm (λ_{exc} = 470 nm) for **MI$_2$H$_2$A$_2$** and 625 nm (λ_{exc} = 560 nm) for **MI$_3$H$_3$A$_3$**. The amount of dye released for each concentration was calculated according to the ratio of the fluorescence of zone B and the fluorescence of the entire strip. As an example, Figure S10 shows the SRB release from **MI$_3$H$_3$A$_3$** in the absence and in the presence of PETN. It is evident that a significant signal in zone B is only registered in the presence of PETN, whereas virtually no fluorescence is observed in zone B in the absence of analyte. The dependence of dye release on the concentration of PETN was found to be similar as in solution. LODs of 0.5 ± 0.2, 0.6 ± 0.2 and 0.4 ± 0.2 ppb were thus determined for the detection of TATP, TNT and PETN, respectively.

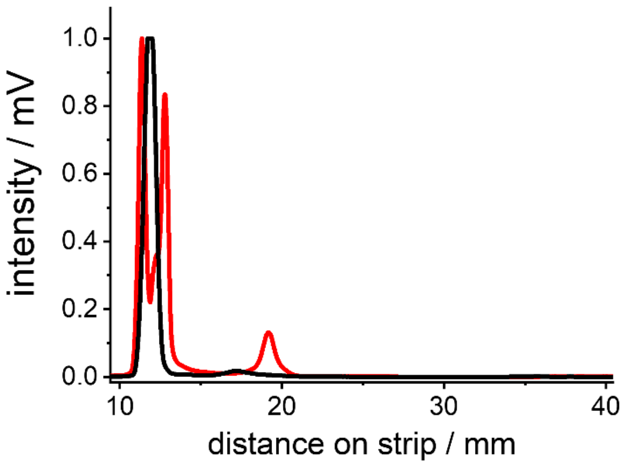

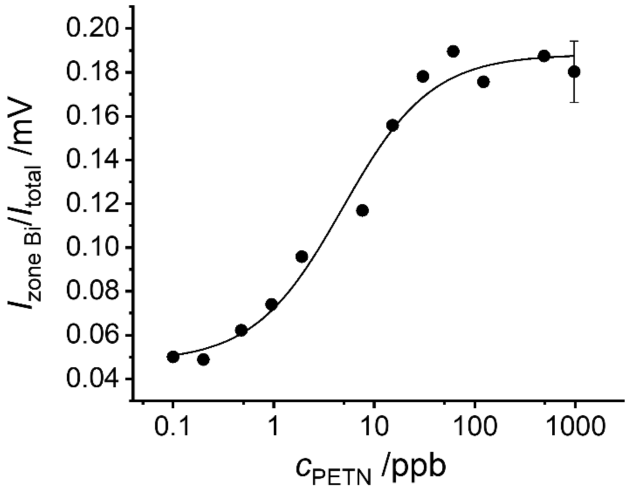

Figure S10. Left: Intensity profile of a representative strip obtained with the fluorescence reader (λ_{exc} = 560 nm, λ_{em} = 625 nm) after the development of the assay showing the fluorescence of the dye SRB = **I$_3$** still contained in the material **MI$_3$H$_3$A$_3$** in zone A at ca. 12 mm and the fluorescence of the dye released from the material and having travelled with the flow in zone B at ca. 19 mm in the absence (black line) and the presence (red line) of 0.1 ppm PETN in PBS buffer (pH 7.4) containing 0.05% Tween and 2.5 % of MeOH. Right: The corresponding release of SRB on several strips containing **MI$_3$H$_3$A$_3$** as a function of the concentration of PETN added. The line exemplifies a four-parametric logistic fit.

For the multiplexing detection of the explosives using a mixture of the materials **MI$_1$H$_1$A$_1$, MI$_2$H$_2$A$_2$** and **MI$_3$H$_3$A$_3$**, first, the chromatographic separation efficiency of the dyes F27, RB3 and SRB had to be assessed for different strip materials. Three different membranes were thus tested, Immunopore RP from Whatman and HF120 and HF135 from Millipore. For that purpose, 1 µL of a mixture of 1 ppm solutions of SRB, F27 and RB3 were deposited at one end of the strip, the strips were dipped into a microwell plate and the flow was left to develop, before taking several pictures with a digital camera under visible and UV light (λ_{exc} = 356 nm) and recording the fluorescence with fluorescence reader. The results are shown in Figure S11, revealing the most efficient separation of the dyes used here was achieved with HF135 from Millipore.

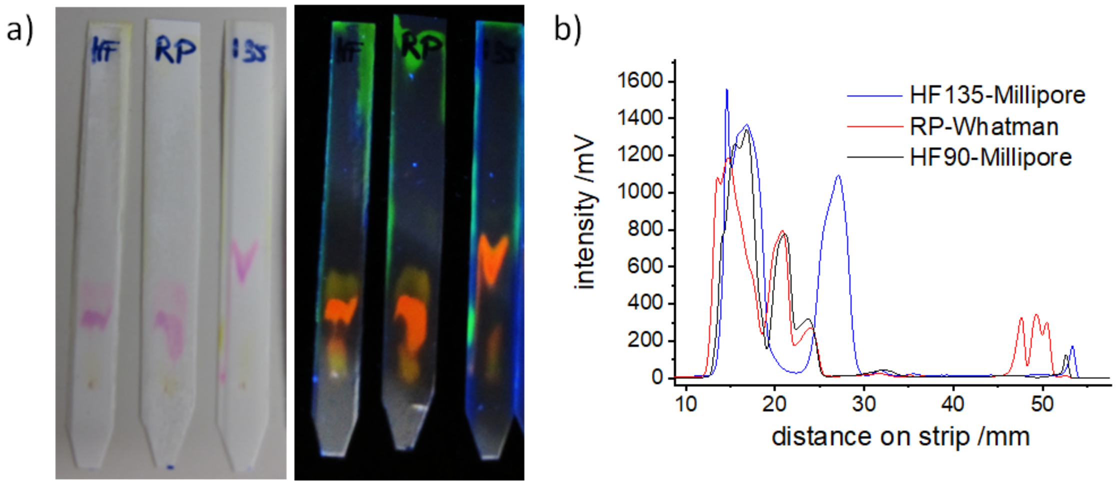

Figure S11 a) Pictures of the different strips tested HF120 (left), RP (middle) HF135 (right) under visible light and UV light (λ_{exc} = 365 nm), showing the spatial separation of the dyes used after the development of the flow in PBS buffer (pH 7.4) containing 0.05% Tween and 2.5 % of MeOH. b) Corresponding intensity profiles obtained with the fluorescence reader (λ_{exc} = 488 nm, λ_{em} = 625 nm) for the three strips after the development of the flow, showing the spatial separation of the three dyes used; F27 travels farthest, SRB second farthest and RB3 remains closest to the spot of deposition.

11 Multiplexing detection of explosives TATP, TNT and PETN

11.1 Smartphone setup as readout device with a single strip

The release of the different dyes was measured with a smartphone setup using two LEDs of 500 nm and 515 nm as excitation sources powered by the smartphone via a USB-OTG link and filtered by a short-pass filter (532 nm) while collecting the emission through a long-pass filter (550 nm) after inserting the developed strip in a 3D-printed, customized holder. The corresponding dye release of the strips containing a mixture of materials **$MI_1H_1A_1$, $MI_2H_2A_2$** and **$MI_3H_3A_3$** is depicted in Figure S12.

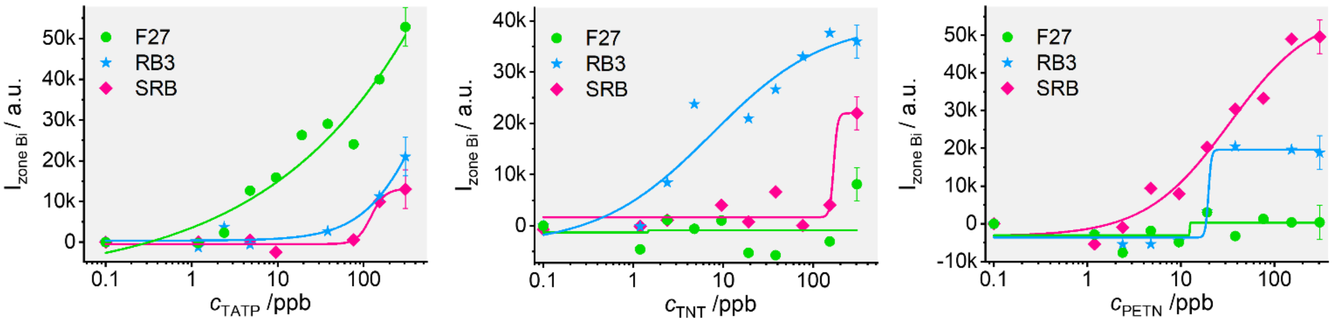

Figure S12. Integrated fluorescence intensity of F27 (**I_1**), RB3 (**I_2**) and SRB (**I_3**) in zones B_i from materials **$MI_1H_1A_1$–$MI_3H_3A_3$** deposited as a mixture in zone A of nitrocellulose strips as a function of the concentration of TATP, TNT or PETN added; the fluorescence of a strip ran only with PBS as a blank was subtracted for removal of background fluorescence. The lines exemplify four-parametric logistic fits.

11.2 Fluorescence reader as readout device with a single strip

The release of the different dyes was also measured with a fluorescence reader, which contained different excitation wavelength/detection filter settings. F27 was recorded at 520 nm (λ_{exc} = 470 nm), RB3 at 625 nm (λ_{exc} = 470 nm) and SRB at 625 nm (λ_{exc} = 560 nm). Corresponding dye release of the strips containing a mixture of materials **$MI_1H_1A_1$, $MI_2H_2A_2$** and **$MI_3H_3A_3$** are depicted in Figure S13.

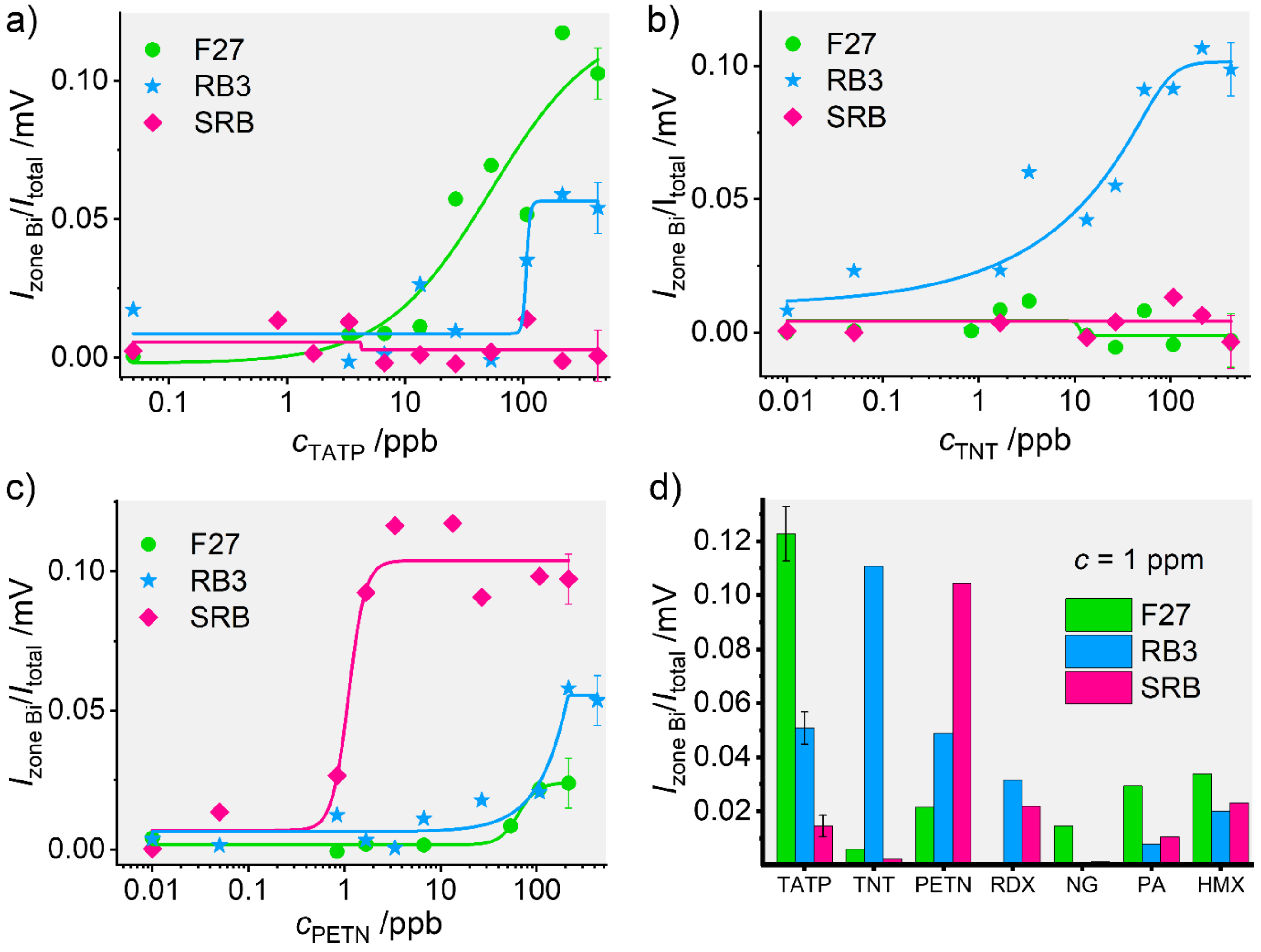

Figure S13. a–c) Release of F27, RB3 and SRB from materials **$MI_1H_1A_1$**, **$MI_2H_2A_2$** and **$MI_3H_3A_3$** deposited on nitrocellulose strips as a function of the concentration of the corresponding explosives TATP, TNT and PETN added, evaluated using a fluorescence reader. d) F27, RB3 and SRB release from materials **$MI_1H_1A_1$**, **$MI_2H_2A_2$** and **$MI_3H_3A_3$** deposited on nitrocellulose strips in the presence of 600 ppb of different explosives. The lines exemplify four-parametric logistic fits.

11.3 Smartphone setup as readout device with patterned strips

Wax-patterning strips with three separate channels branching out from a single sample reservoir and containing the sensing hybrids materials were prepared. 30 µl of a solution containing different concentrations of buffer employed, TATP, TNT or PETN were deposited on the sample deposition zone and then fluorescence intensity of dye released on the corresponding B zones was evaluated taking pictures of the strips with a smartphone. Figure S14 shows differences in fluorescence intensity of dye released as a function of the concentration of different explosives used.

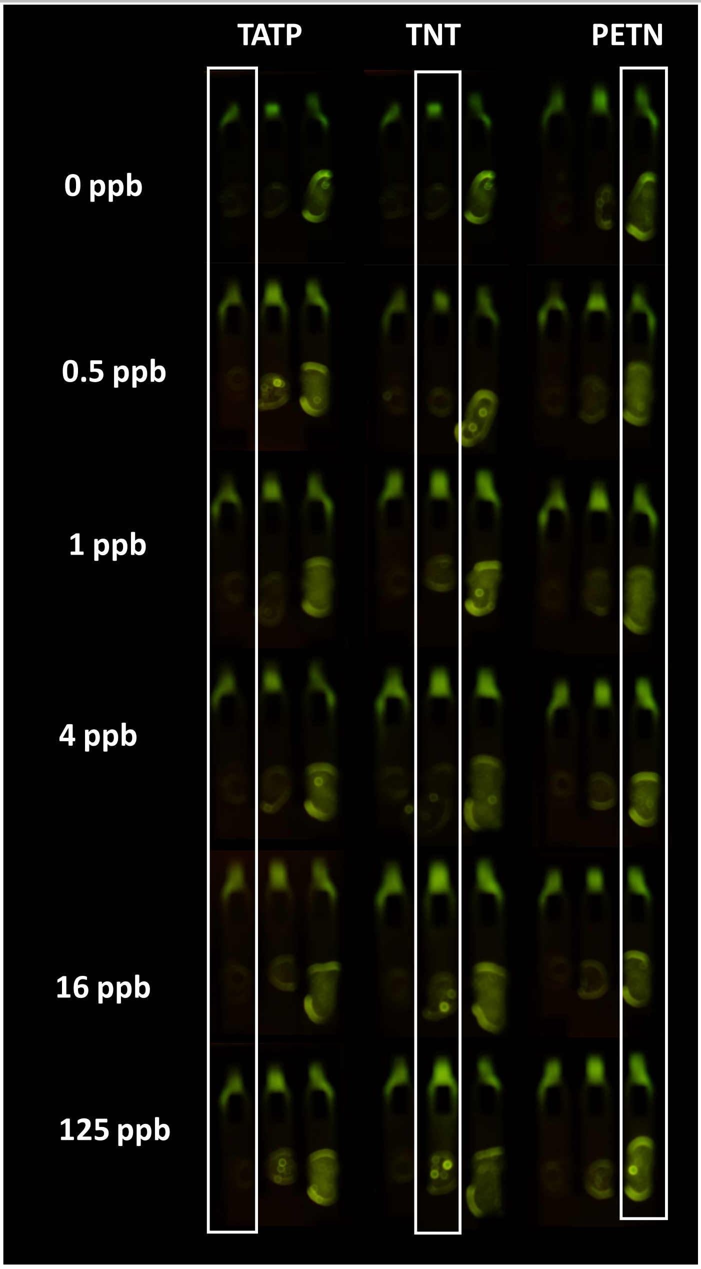

Figure S14. Collage of the photographs corresponding to the plots in Figure 5, shot under proper light excitation and showing the release of F27 (**I_1**) from materials **$MI_1H_1A_1$**, **$MI_1H_2A_2$** and **$MI_1H_3A_3$** in the presence of various amounts of TATP, TNT or PETN.

Following a similar procedure as described, several strips were exposed to different solutions of a mixture of 3 explosives tested (Figure S15, experiment 2) or a mixture of only two explosives (Figure S15, experiments 3-6), with the aim to evaluate the possible interference on the sensing materials. Strips exposed only to buffered media were also evaluated as reference (Figure S15, experiment 1). As can be seen in Figure 15, fluorescence intensity on the respective zones Bi were increased only when the corresponding explosive was present on the buffered media, showing no fluorescence enhancement on the channel corresponding to the explosive absent.

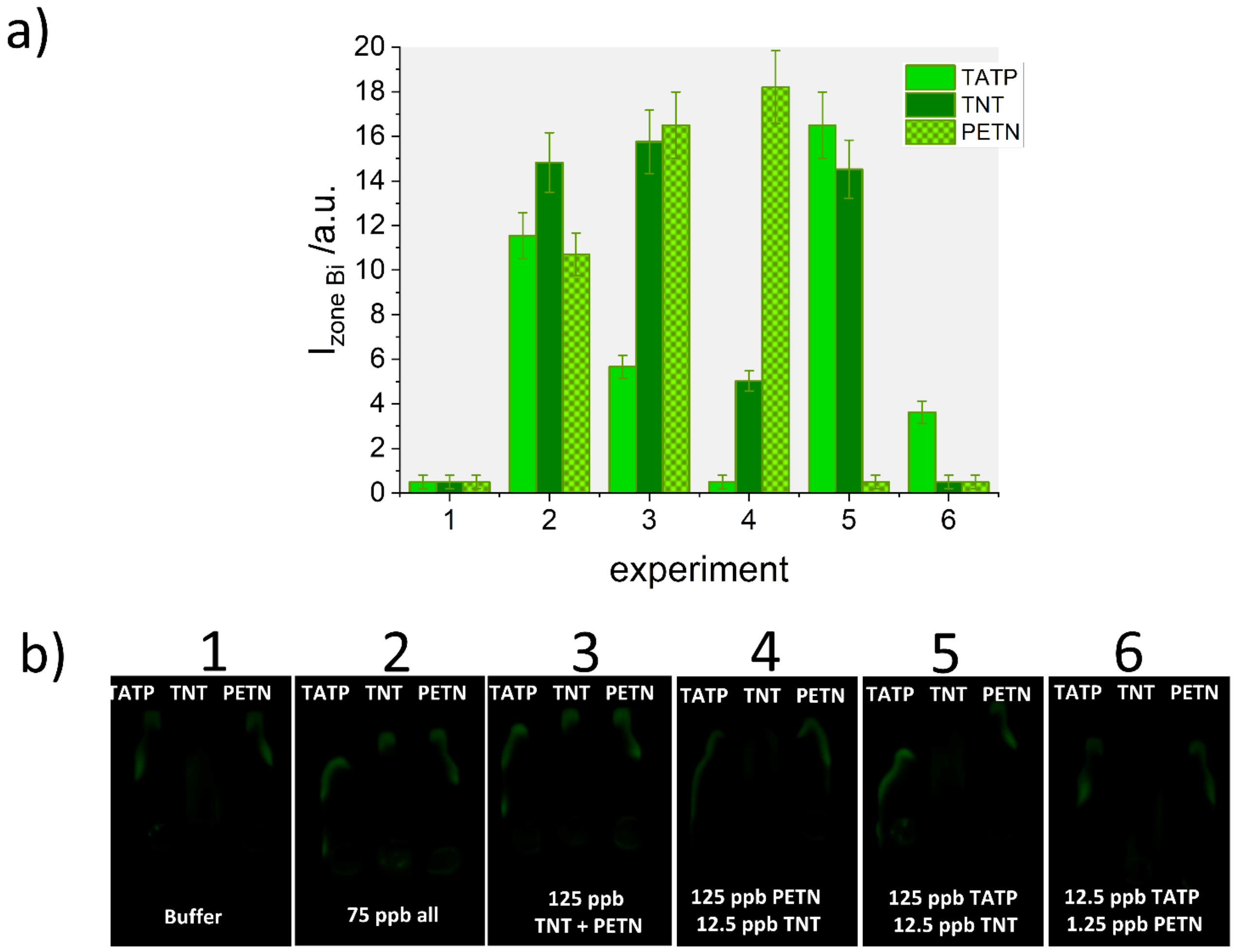

Figure S15. a) F27 dye released from materials **$MI_1H_1A_1$**, **$MI_1H_2A_2$** and **$MI_1H_3A_3$** in the presence of several mixtures of TATP, TNT or PETN b) Corresponding images of the zones A and B of the corresponding strips as registered with the smartphone, shot under proper light excitation and showing the release of F27 (**I_1**) from materials in the presence of various amounts of TATP, TNT or PETN.

12 Additional notes on analytical performance

12.1 Limits of detection

For the experiments in suspension, the fluorescence emission of the supernatants of the suspensions of the materials in the presence of different concentrations of explosives were recorded, and the values of the fluorescence enhancement observed during the titrations were plotted vs. analyte concentration. These curves were fitted to a four-parameter logistic fitting function (equation S1).[5]

$$\frac{I}{I_0} = \frac{F_1 - F_2}{\left(1 + \frac{x}{x_0}\right)^P} + F_2 \qquad \text{(S1)}$$

Here, F_1 and F_2 correspond to the minimum and maximum enhancement of fluorescence observed, and *p* corresponds to the slope of the sigmoidal curve. Limits of detection (LODs), which describe the smallest concentration of analyte that can be reliably detected, were derived calculating in a first instance the Limits of Blank (LOBs; describing the smallest concentration of analyte that can be observed) and calculating the corresponding concentration of the signal as follows:[6]

LOB = mean blank + 1.645(σ_{blank})

LOD = LOB + 1.645($\sigma_{low\ concentration\ sample}$)

Generally, three repeat experiments were carried out for both the kinetics and the concentration-dependent measurements.

Measurement uncertainties were derived as detailed in ref. [7].

12.2 Repeatability

Regarding the strip-to-strip repeatability, within the same batch, repeat errors of 4-6% were found between replicates, the main problem being the slightly different flow behavior on the nitrocellulose membranes, having a certain impact on the area of the dye in the detection zone.

12.3 Stability

When kept under normal laboratory conditions (in dry state at 4°C under air atmosphere) in a conventional refrigerator, the materials are stable for at least 3 months. After that period, the amount of dye that could be maximally released decreased, and a certain blank release was also noticed. When the material is kept on the strips (in dry state at 4°C under air atmosphere), this combination can be stored up to 2 months in a refrigerator. Afterwards, flow on the strips is becoming slower, possibly due to aggravated wetting. However, we tentatively assume that the stability of the material, as well as the final assay strip, can be improvable when the strips would be packaged under CA (controlled atmosphere) conditions.

12.4 Cut-off values

Simple YES/NO responses are the preferred format in which results of rapid on-site tests are commonly delivered to the user, because decision making in the application scenarios usually has to be fast. Commercial hand-held sensors such as the Fido X3, which can detect various classes of explosives (https://www.flir.com/products/fido-x3/), are thus also providing only concise YES/NO information to the use. The evaluation of the analytical results is usually done on the basis of a threshold or cut-off value, in the case of the Fido X3 for instance also amounting to <5%.

12.5 Previously reported immunoassays for explosives detection

Table S2. Representative selection of immunoassays for explosives detection published in the literature.

No.	Analyte	Type of IA	Format [a]	Detection [b]	LOD /ppb	Range	Time	Multiplexing and/or hand-held	Ref.
1	TNT	competitive	ELISA	colorimetric	2	2–30,000	hours	no	[8]
2	TNT	competitive	ELISA	colorimetric	0.06	0.06–2	2 h	no	[9]
3	TNT	competitive, indirect	ELISA	CL	0.2	0.2–50	hours	no	[10]
4	TNT	competitive	ELISA	colorimetric	0.0006	0.005–150	hours	no	[2b]
5	RDX	competitive	ELISA	colorimetric	0.3	0.3–6	hours	no	[11]
6	TATP	competitive	ELISA	colorimetric	65	65–10,000	hours	no	[12]
7	TATP	competitive	ELISA	colorimetric	0.01	30–500	hours	no	[2a]
8	PETN	competitive	ELISA	colorimetric	0.5	1–1,000	hours	no	[2c]
9	TNT	displacement	reader-based	fluorometric	0.5	0.5–100	30 min	no	[13]
10	TNT	displacement	FIA	fluorometric	2.5	20–1,200	few min.	no	[14]

No.	Analyte	Type of IA	Format [a]	Detection [b]	LOD /ppb	Range	Time	Multiplexing and/or hand-held	Ref.
11	RDX	displacement	FIA	fluorometric	15	18–300	few min.	no	[15]
12	TNT or RDX	displacement	FIA	fluorometric	<1	1–600	few min.	no	[16]
13	TNT	displacement, reversed	FIA	fluorometric	2.5	20–10,000	10 min	no	[17]
14	TNT	displacement, reversed	microcapillary-based IA	fluorometric	0.25	1–1000	5 min	no	[18]
15	TNT	displacement	microfluidic chip-based IA	fluorometric	1–10	3 orders of magnitude	1.5–2 h	no	[19]
16	TNT	competitive	electrophoretic chip-based IA	fluorometric	1	1–300	50 min	no	[20]
17	TNT	competitive	electrophoretic chip-based IA	fluorometric	1	1–300	50 min	no	[21]
18	TNT	competitive, indirect	gold chip-based IA	SPR	0.09	0.09–1000	25 min	no	[22]
19	TNT	competitive, indirect	gold chip-based IA	SPR	0.002	0.008–30	5 min	no	[23]
20	TNT	competitive, indirect	gold chip-based IA	SPR	0.008	0.008–30	5 min	no	[24]
21	TNT	competitive, indirect	gold chip-based IA	SPR	0.15	0.5–7	10 min	no	[25]
22	TNT	competitive, indirect	gold chip-based IA	SPR	0.01	0.01–100	few min.	no	[26]
23	TNT	displacement	gold chip-based IA	SPR	0.08		10s mins	no	[27]
24	TNT	displacement	gold chip-based IA	SPR	0.4	1–100	10s mins	no	[28]
25	TNT	displacement	QCM-based IA	frequency shift	0.1	0.1–10,000	10 min	no	[29]
26	TNT	competitive	LF-IA	colorimetric	1000	not reported	5 min	hand-held	[10]
27	TNT	displacement	bead-based cytometer IA	fluorometric	0.1	1–10,000	30 min	potentially multiplexable	[30]
28	TNT	competitive	bead-based cytometer IA	fluorometric	1	1–1000	40 min	potentially multiplexable	[31]
29	TNT	competitive	bead-based cytometer IA	fluorometric	1	100–10,000	40 min	potentially multiplexable	[32]
30	TATP and TNT and PETN	displacement	gAID	fluorometric	ca. 0.5	from 1–15 to 5–300	5 min	both	this work

[a] ELISA = enzyme linked immunosorbent assay, FIA = flow immunoassay, IA = immunoassay, QCM = quartz crystal microbalance, LF-IA = lateral flow immunoassay. [b] CL = chemiluminescence, SPR = surface plasmon resonance

References

[1] J. G. Tatake, M. M. Knapp, C. Ressler, *Bioconjugate Chem.* **1991**, *2*, 124-132.

[2] a) M. A. Walter, U. Panne, M. G. Weller, *Biosensors* **2011**, *1*, 93-106; b) S. Ramin, M. G. Weller, *J. Mol. Recognit.* **2012**, *25*, 89-97; c) A. Hesse, M. Biyikal, K. Rurack, M. G. Weller, *J. Mol. Recognit.* **2016**, *29*, 88-94; d) E. Climent, D. Groninger, M. Hecht, M. A. Walter, R. Martínez-Máñez, M. G. Weller, F. Sancenón, P. Amorós, K. Rurack, *Chem. Eur. J.* **2013**, *19*, 4117-4122.

[3] E. Climent, L. Mondragon, R. Martinez-Manez, F. Sancenon, M. Dolores Marcos, J. Ramon Murguia, P. Amoros, K. Rurack, E. Perez-Paya, *Angew. Chem. Int. Ed.* **2013**, *52*, 8938-8942.

[4] E. Costa, E. Climent, K. Gawlitza, W. Wan, M. G. Weller, K. Rurack, *J. Mater. Chem. B* **2020**, *8*, 4950-4961.

[5] Y. Xiang, J. Donley, E. Seletskaia, S. Shingare, J. Kamerud, B. Gorovits, *AAPS J.* **2018**, *20*, Art. No. 45.

[6] D. A. Armbruster, T. Pry, *Clin. Biochem. Rev.* **2008**, *29 Suppl 1*, S49-S52.

[7] E. Costa, E. Climent, S. Ast, M. G. Weller, J. Canning, K. Rurack, *Analyst* **2020**, *145*, 3490-3494.

[8] P. Julicher, E. Mussenbrock, R. Renneberg, K. Cammann, *Anal. Chim. Acta* **1995**, *315*, 279-287.

[9] A. Zeck, M. G. Weller, R. Niessner, *Fresenius J. Anal. Chem.* **1999**, *364*, 113-120.

[10] S. Girotti, S. A. Eremin, A. Montoya, M. J. Moreno, P. Caputo, M. D'Elia, L. Ripani, F. S. Romolo, E. Maiolini, *Anal. Bioanal. Chem.* **2010**, *396*, 687-695.

[11] D. O. Ulaeto, A. P. Hutchinson, S. Nicklin, *Monoclonal Antibodies Immunodiagn. Immunother.* **2015**, *34*, 225-227.

[12] M. A. Walter, D. Pfeifer, W. Kraus, F. Emmerling, R. J. Schneider, U. Panne, M. G. Weller, *Langmuir* **2010**, *26*, 15418-15423.

[13] E. R. Goldman, G. P. Anderson, N. Lebedev, B. M. Lingerfelt, P. T. Winter, C. H. Patterson, J. M. Mauro, *Anal. Bioanal. Chem.* **2003**, *375*, 471-475.

[14] J. P. Whelan, A. W. Kusterbeck, G. A. Wemhoff, R. Bredehorst, F. S. Ligler, *Anal. Chem.* **1993**, *65*, 3561-3565.

[15] J. C. Bart, L. L. Judd, A. W. Kusterbeck, *Sens. Actuators B* **1997**, *39*, 411-418.

[16] S. Y. Rabbany, W. J. Lane, W. A. Marganski, A. W. Kusterbeck, F. S. Ligler, *J. Immunol. Methods* **2000**, *246*, 69-77.

[17] T. M. Green, P. T. Charles, G. P. Anderson, *Anal. Biochem.* **2002**, *310*, 36-41.

[18] P. T. Charles, J. G. Rangasammy, G. P. Anderson, T. C. Romanoski, A. W. Kusterbeck, *Anal. Chim. Acta* **2004**, *525*, 199-204.

[19] P. T. Charles, A. A. Adams, P. B. Howell, S. A. Trammell, J. R. Deschamps, A. W. Kusterbeck, *Sensors* **2010**, *10*, 876-889.

[20] A. Bromberg, R. A. Mathies, *Anal. Chem.* **2003**, *75*, 1188-1195.

[21] A. Bromberg, R. A. Mathies, *Electrophoresis* **2004**, *25*, 1895-1900.

[22] D. R. Shankaran, K. V. Gobi, T. Sakai, K. Matsumoto, K. Toko, N. Miura, *Biosens. Bioelectron.* **2005**, *20*, 1750-1756.

[23] D. R. Shankaran, T. Kawaguchi, S. J. Kim, K. Matsumoto, K. Toko, N. Miura, *Anal. Bioanal. Chem.* **2006**, *386*, 1313-1320.

[24] T. Kawaguchi, D. R. Shankaran, S. J. Kim, K. V. Gobi, K. Matsumoto, K. Toko, N. Miura, *Talanta* **2007**, *72*, 554-560.

[25] P. Singh, T. Onodera, Y. Mizuta, K. Matsumoto, N. Miura, K. Toko, *Sens. Mater.* **2007**, *19*, 261-273.

[26] T. Kawaguchi, D. R. Shankaran, S. J. Kim, K. Matsumoto, K. Toko, N. Miura, *Sens. Actuators B* **2008**, *133*, 467-472.

[27] Y. Mizuta, T. Onodera, P. Singh, K. Matsumoto, N. Miura, K. Toko, *Biosens. Bioelectron.* **2008**, *24*, 191-197.

[28] T. Onodera, Y. Mizuta, K. Horikawa, P. Singh, K. Matsumoto, N. Miura, K. Toko, *Sens. Mater.* **2011**, *23*, 39-52.

[29] J. Wang, M. AlMakhaita, S. L. Biswal, L. Segatori, *J. Biosens. Bioelectron.* **2012**, *3*, 1-7.

[30] G. P. Anderson, S. C. Moreira, P. T. Charles, I. L. Medintz, E. R. Goldman, M. Zeinali, C. R. Taitt, *Anal. Chem.* **2006**, *78*, 2279-2285.

[31] G. P. Anderson, J. D. Lamar, P. T. Charles, *Environ. Sci. Technol.* **2007**, *41*, 2888-2893.

[32] G. P. Anderson, E. R. Goldman, *J. Immunol. Methods* **2008**, *339*, 47-54.

Author Contributions

E.C. and K.R. with R.M.M. conceived the experiments. E.C. with M.B. and D.G. performed the experiments. M.G.W. curated the immunoassay data, E.C. and K.R. with M.G.W. and R.M.M. prepared the manuscript. All authors discussed the results and commented on the manuscript.
